# Antagonistic Potential of Fluorescent Pseudomonads Colonizing Wheat Heads Against Mycotoxin Producing Alternaria and Fusaria

**DOI:** 10.3389/fmicb.2018.02124

**Published:** 2018-09-10

**Authors:** Thomas Müller, Silke Ruppel, Undine Behrendt, Peter Lentzsch, Marina E. H. Müller

**Affiliations:** ^1^Leibniz Centre for Agricultural Landscape Research, Müncheberg, Germany; ^2^Berlin-Brandenburg Institute of Advanced Biodiversity Research, Berlin, Germany; ^3^Leibniz Institute of Vegetable and Ornamental Crops, Großbeeren, Germany

**Keywords:** agriculture, wheat, plant microbiota, natural control, *Pseudomonas*, *Alternaria*, *Fusarium*, mycotoxins

## Abstract

Natural control of phytopathogenic microorganisms is assumed as a priority function of the commensal plant microbiota. In this study, the suitability of fluorescent pseudomonads in the phyllosphere of crop plants as natural control agents was evaluated. Under field conditions, ears of winter wheat were found to be colonized with high consistency and at a high density by pseudomonads at the late milk dough stage. Isolates of these bacteria were evaluated for their potential to protect the plants from phytopathogenic *Alternaria* and *Fusarium* fungi. More *Pseudomonas* isolates were antagonistically active against alternaria than against fusaria in the dual culture test. The alternaria responded species-specifically and more sensitively to bacterial antagonism than the strain-specific reacting fusaria. A total of 110 randomly selected *Pseudomonas* isolates were screened for genes involved in the biosynthesis of the antibiotics 2,4-diacetylphloroglucinol, phenazine-1-carboxylic acid, pyoluteorin, and pyrrolnitrin. The key gene for production of the phloroglucinol was found in none of these isolates. At least one of the genes, encoding the biosynthesis of the other antibiotics was detected in 81% of the isolates tested. However, the antagonistic effect found in the dual culture assay was not necessarily associated with the presence of these antibiotic genes. Wheat grains as natural substrate were inoculated with selected antagonistic *Pseudomonas* isolates and *Alternaria* and *Fusarium* strains, respectively. The fungal growth was only slightly delayed, but the mycotoxin production was significantly reduced in most of these approaches. In conclusion, the distribution of phytopathogenic fungi of the genera *Alternaria* and *Fusarium* in the field is unlikely to be inhibited by naturally occurring pseudomonads, also because the bacterial antagonists were not evenly distributed in the field. However, pseudomonads can reduce the production of *Alternaria* and *Fusarium* mycotoxins in wheat grains and thus have the potential to improve the crop quality.

## Introduction

Bacteria of the genus *Pseudomonas* are commonly found among the predominant genera in plant microbiomes, both in the rhizosphere (Bakker et al., 2013; Donn et al., 2015) and in the phyllosphere (Vorholt, 2012; Bulgarelli et al., 2013; Rastogi et al., 2013), irrespective of the host plant species. Species of this genus are characterized by a great diversity of ecological, metabolic, and biochemical capabilities and activities (Palleroni, 2005). In plant-association, these capabilities are manifested in saprophytic, pathogenic or plant growth-promoting modes of action. Plant growth-promoting pseudomonads are directly capable of promoting plant growth through the synthesis of growth-stimulating phytohormones or indirectly through enhanced pathogen protection (Haas and Défago, 2005; Wu et al., 2012; Schlaeppi and Bulgarelli, 2015). These activities are attributable to a group of non-phytopathogenic, non-necrogenic species of pseudomonads secreting a yellow-green fluorescent pigment, the siderophore pyoverdine (Meyer et al., 2002), which supports the iron acquisition of bacterial cells (Loper et al., 2012).

The ecology of plant-associated fluorescent pseudomonads with special attention to their specific role in the natural control of phytopathogens in soils and the rhizosphere has already been reviewed (Raaijmakers et al., 2002; Haas and Défago, 2005; McSpadden Gardener, 2007). The suppression of soil-borne pathogens by pseudomonads is mainly linked to their secretion of secondary metabolites, such as phloroglucinols, phenazines, pyoluteorin, pyrrolnitrin, and hydrogen cyanide (Haas and Défago, 2005). Haas and Défago (2005) also described an antimicrobial effect by the extracellular diffusible siderophore pyoverdine, which lends the pseudomonads their fluorescence. Siderophores of this type may function as contingent antibiotics and contribute to disease suppression by depriving pathogens of iron.

The antagonistic activity of these bacteria was also the subject of research in the phyllosphere. Alimi et al. (2012) and Yoshida et al. (2012) reported on the inhibition potential of fluorescent pseudomonads isolated from wheat heads against the fungal pathogens of Fusarium head blight (FHB). Preliminary results of a recent study in our laboratory confirmed these findings: a high proportion of *Pseudomonas* strains isolated from wheat leaves were antagonistically active against fusaria and alternaria, which may cause FHB and the so-called “black point” disease, respectively (Müller et al., 2016).

Fusarium head blight as well as black point are very common diseases in wheat in the temperate climate of Central Europe. They cause heavy losses in yield and quality (Windels, 2000; Eggert et al., 2011). The main causative agents of FHB are *Fusarium graminearum* [teleomorph *Gibberella zeae* (Schwein) Petch] and *Fusarium culmorum* [(W.G. Smith) Sacc (teleomorph unknown)] (Bottalico and Perrone, 2002; Yang et al., 2013), whereas those of the black point disease are *Alternaria alternata* (Fr.) Keissler and *Alternaria tenuissima* ([Kunze ex Nees et T. Nees: Fries] Wiltshire). These *Alternaria* fungi occur ubiquitously on wheat leaves and ears, and therefore, the black point disease is widespread in humid and semiarid regions around the world (reviewed in Vučković et al., 2012). In addition to yield losses and reduced grain quality, these *Alternaria* as well as *Fusarium* species are known to produce mycotoxins, which are harmful to both humans and livestock (Logrieco et al., 2009; McMullen et al., 2012). Under the climatic condition of Central Europe, winter wheat is exposed to both fusaria and alternaria and can be contaminated by their mycotoxins (Bottalico and Perrone, 2002; Müller and Korn, 2013).

The above mentioned studies on suppression of soil-borne pathogens by naturally occurring pseudomonads (Raaijmakers et al., 2002; Haas and Défago, 2005; McSpadden Gardener, 2007) suggested that crop losses caused by phytopathogenic fungi can be kept in reasonable limits by a natural control, i.e., a form of biological control, in which the number of pathogenic individuals in a population is reduced by a natural enemy without human intervention (Bale et al., 2008). It is claimed that the plant microbiome provides a wide range of potential antagonists for the protection against phytopathogens (Bulgarelli et al., 2013; Schlaeppi and Bulgarelli, 2015; Gopal and Gupta, 2016). The overarching question of our present study is, whether this is also true for fluorescent pseudomonads that colonize the phyllosphere of crop plants. Therefore, the present study aimed to check and deepen the role of pseudomonads as natural control agents. We addressed the question of whether fluorescent pseudomonads in the phyllosphere of wheat have an antagonistic potential against *Alternaria* and *Fusarium* fungi and besides that, whether they can affect the production of mycotoxins by these fungi as well. For this reason, a representative number of *Pseudomonas* isolates from wheat ears were tested for antagonistic activity *in vitro* against alternaria and fusaria. Additionally, a screening for genes coding for the biosynthesis of antibiotics 2,4-diacetylphloroglucinol (DAPG), phenazine-1-carboxylic acid (PCA), pyoluteorin, and pyrrolnitrin in selected isolates should provide indications of possible mechanisms of bacterial antagonistic activity. Finally, fungal growth and mycotoxin production were investigated in wheat grains co-inoculated with *Alternaria* or *Fusarium* strains and antagonistic *Pseudomonas* isolates.

## Materials and Methods

### Strains

A series of 10 *Alternaria* and 10 *Fusarium* isolates including the reference strains *A. alternata* CABI 353822, *A. tenuissima* CABI 352931, and *A. infectoria* CBS 120149, supplemented by *Ulocladium* spec. 219 were used as indicators in the dual culture assay. The non-reference strains had previously been isolated from wheat plants (Korn et al., 2011; Müller et al., 2012). These fungi are stored as single-spore-cultures in the culture collection of microorganisms of the working group “Fungal Interactions” at the Leibniz Centre of Agricultural Landscape Research Müncheberg. The root-born strain *Pseudomonas protegens* CHA0^T^ (Ramettea et al., 2011) (formerly *P. fluorescens* CHA0), which is able to produce various antagonistic secondary metabolites (Natsch et al., 1994) was used as a reference strain for the detection of genes encoding the biosynthesis of DAPG and pyrrolnitrin. In addition, *Pseudomonas libanensis* 9 and *Pseudomonas rhodesiae* 188, which have already been taxonomically characterized and tested for antagonistic activity (Müller et al., 2016), were used as additional reference strains in antagonistic tests and taxonomic identification.

### Sampling

Ears of winter wheat (cultivar Meister) were sampled in three fields in the Northeast Lowlands of Germany, the Uckermark region (GPS coordinates of field 1: X_408066.50977/Y_5912493.88704; of field 2: X_420313.84817/Y_5916253.10291; of field 3: X_408812.71676/Y_5909328.33314), at the late milk/early dough stage Z76-Z83 (Zadoks et al., 1974) in 2015 and 2016. At each of 151 points at a distance of at least 50 m from each other, 10 ears were aseptically removed from neighboring plants and transported on ice in sterile plastic bags. These bulk samples were stored for a maximum of 4 weeks at -20°C until processing as a single sample.

### Enumeration and Isolation of Bacteria

An amount of 8–10 g fresh matter (FM) of ears was immersed in 100 mL sterile aqua dest. and treated for 2 min at high speed in the Stomacher 400 Circulator (Seward Ltd., Thetford, United Kingdom). The suspensions were diluted with quarter-strength Ringer’s solution and spread-plated onto King’s B agar (ROTH, Karlsruhe, Germany). After incubation at 25°C for 5 days, colonies fluorescing at 366 nm excitation wavelength were enumerated. From each of 22 selected samples, about 30 fluorescent colonies were picked up from the agar surface. The isolates were purified by fractionated spreading twice on the same agar, re-cultivated in Standard I Nutrient Broth (MERCK, Darmstadt, Germany) overnight, transferred in Cryobank tubes (Mast Diagnostica, Reinfeld, Germany) and stored at -20°C.

### Dual Culture Test

The antagonistic activity of all *Pseudomonas* isolates against phytopathogenic *Fusarium* and *Alternaria* strains and the saprophytic *Ulocladium* spec. 219 (*U*219) was tested by dual culturing of the bacteria and fungi on Potato Dextrose Agar (ROTH, Karlsruhe, Germany) adjusted to pH 6.5. On each agar plate, four bacterial isolates pre-cultivated overnight in Standard I Nutrient Broth (MERCK, Darmstadt, Germany) were spread in straight lines of 30 mm and at a distance of 35 mm from the center of a 90 mm Petri dish. After 2 days of incubation at 25°C, an agar plug of 8 mm diameter with growing mycelium of one of the fungal indicator strains was placed in the center of the agar plate. The plates were incubated at 25°C until the growing fungal colony on a control plate without bacterial inoculation reached a radius of 35 mm. Criterion for antagonistic activity was a distance between the edge of the fungal colony and the bacterial smear (the inhibition zone) of at least 3 mm. Antagonistic activity was quantified as none (0–3 mm inhibition zone), low (3–5 mm), moderate (5–10 mm), and strong (>10 mm) (**Supplementary Figure S1**). The tests were repeated twice.

### Taxonomic Characterization of *Pseudomonas* Isolates by MALDI-TOF MS

A total of 145 isolates, including those without, with low and high activity in the dual culture assay, were selected for taxonomic characterization. The preparation of isolates for MALDI-TOF MS analysis and the procedure of spectra generation were described in detail by Müller et al. (2016). The processed spectra were compared to each other and with spectra in the MALDI Biotyper^TM^ reference database (version 3.0, Bruker Daltonics, Bremen, Germany). The closeness of the match to the reference spectra of the database or between spectra of the isolates was reflected in a score value that was calculated during the matching process. Score values were grouped into different categories: >2.3 – highly probable species identification, 2.0 to 2.299 – secure genus identification and probable species identification, and 1.7 to 1.999 – probable genus identification. Isolates that originate from the same sample displaying score values >2.3 between their spectra and carry the same antibiotic encoding genes were reduced to one isolate per sample to exclude clonality. On this basis, the number of isolates representing a strain was reduced to 110.

### Screening for Genes Encoding the Biosynthesis of Known Antibiotics

The same isolates that were taxonomically characterized were also tested for the presence of genes involved in the biosynthesis of antibiotics: the *phlD* gene for the production of DAPG, the *phz* gene for PCA, the *pltB* gene for pyoluteorin, and the *prnC* gene for pyrrolnitrin. A 2-mL sample of bacteria grown in Standard I Nutrient Broth (MERCK, Darmstadt, Germany) for 24 h at 25°C was centrifuged (14.400 rpm for 5 min at 4°C). The supernatant was discarded, the cells in the pellet were disrupted by means of a High-speed benchtop homogenizer MP FastPrep 24 (MP Biomedicals Germany GmbH, Eschwege, Germany), and DNA was extracted using the DNeasy Plant Mini Kit (QIAGEN GmbH, Hilden, Germany) according to the manufacturer’s instruction for use. The *phlD*, *phz, pltB*, and *prnC* genes were detected by quantitative real-time PCR (qPCR) with the primer pairs B2BF/BPR4, PCA2a/PCA3b, PltBf/PltBr, and Prncf/Prncr, respectively, described by Shirzad et al. (2012). The PCR product size and purity were checked using the melting profile and agarose gel electrophoresis.

### Antagonism Test on Natural Substrate

#### The Test Procedure

An amount of 8 g of wheat grains, moistened with 10 mL of distilled water and autoclaved three times, in a 100 mL Erlenmeyer flask was successively inoculated with one bacterial isolate and one fungal strain and incubated at 25°C. Controls to observe undisturbed fungal growth were prepared by inoculation of only the fungal suspension with the same density as described below. The bacterial and fungal growth as well as the production of mycotoxins was analyzed immediately after inoculating the fungi, and 7 as well as 13 days after. These analyses were performed in quintuplicate, i.e., each inoculation variant was placed into five parallel Erlenmeyer flasks for analyses after each incubation interval.

#### Preparation of Bacterial Inoculants

The *Pseudomonas* isolates 279, 342, 423, 491, and 500 used in this test were pre-cultured in Standard I Nutrient Broth (MERCK, Darmstadt, Germany) overnight at 25°C. A suspension density of 6 × 10^6^ cells mL^-1^ was adjusted with sterile 1/4 strength Ringer’s solution by means of a Thoma counting chamber of 0.01 mm depth (Poly-Optik GmbH, Bad Blankenburg, Germany). The addition of 250 μL of this suspension to the wheat grains in the Erlenmeyer flask resulted in an inoculation density of approximately 1 × 10^5^ cells g^-1^ of grain FM.

#### Preparation of Fungal Inoculants

The fungal inoculum was added 2 days after incubation of the grains inoculated with the bacteria. The strains *F. culmorum* 13 and *F. graminearum* 23 were pre-cultivated on Synthetic Nutrient Agar (Nirenberg, 1976). *A. tenuissima* 220 was grown on Potato Dextrose Agar (ROTH, Karlsruhe/Germany). After pre-incubation of the fungi for 7 days at 25°C in the dark, an incubation followed under mixed black light (near UV, emission 310–360 nm) and artificial daylight with a 12 h:12 h light:dark cycle for 5 days. Fungal spores were harvested by washing the culture surface with sterile 1/4 strength Ringer’s solution. A Thoma counting chamber of 0.1 mm depth (Poly-Optik GmbH, Bad Blankenburg, Germany) was used to adjust a spore density of 6 × 10^4^ cells mL^-1^ in 1/4 strength Ringer’s solution. By adding 250 μL of this suspension, an inoculation titre of approximately 1 × 10^3^ spores g^-1^ of grains (FM) was achieved. To prepare untreated controls, 250 μL of sterile 1/4 strength Ringer’s solution were added twice to the grains.

#### Bacterial Growth Quantification

Bacteria were enumerated as described above (Enumeration and isolation of bacteria).

#### Fungal Growth Quantification

A sub-sample of about 1 g of the fungus-bacterium-grain mixture was taken out from the Erlenmeyer flask to prepare the qPCR analyses. These sub-samples were dried at 60°C for 24 h and ground to fine flour (Mixer Mill MM 200, Retsch GmbH, Haan, Germany) for DNA extraction.

Genomic DNA was extracted from 50 mg DM wheat grain mixture using the DNeasy Plant Mini kit (QIAGEN GmbH, Hilden, Germany). After 16 h of lysis in a rotary incubator (ENVIRO-Genie, Scientific Industries Inc., Bohemia, NY, United States) at 65°C and at a rotational speed of 25 rpm, reproducible amounts of fungal DNA were extracted from the cereal grain matrix. The total amounts of purified DNA were assessed using a NanoDrop 1000 micro-volume spectrophotometer (NanoDrop Products, Wilmington, DE, United States).

The qPCR assay was conducted in polypropylene 96-well plates using the QuantStudio^TM^ 12K Flex Real-Time PCR System (Life Technologies, Grand Island, NY, United States). Each of the 20 μL reaction approaches contained 4 μL of 5× HOT FIREPol^®^ Probe Universal qPCR Mix ROX (Solis BioDyne, Tartu, Estonia), 0.30 μl of the corresponding forward and reverse primers and 0.66 μL of FAM-labeled probe (biomers.net, Ulm, Germany), each component diluted to 10 pM, as well as 13.74 μL of Millipore H_2_O and 1 μL of template DNA. Standard curves were generated by dilution series of DNA from *A. tenuissima* GH50t for the quantification of alternaria (efficiency >0.91 and *R*^2^ > 0.998) and of DNA from *F. graminearum* 486 for the quantification of fusaria (efficiency >0.89 and *R*^2^ > 0.96). The fungi are stored in the culture collection of microorganisms of the working group “Fungal Interactions” at the Leibniz Centre of Agricultural Landscape Research Müncheberg.

Primers and probes described by Grube et al. (2015) were used for detection of different *Alternaria* species-groups. *In silico* tests of DNA sequences (software package DNA star, DNASTAR, Inc., Madison, WI, United States) resulted in the detection of all genetically defined species of the *Alternaria*-groups – *A. alternata*, *A. tenuissima* and *A. arborescens* – according to Lawrence et al. (2013) and Woudenberg et al. (2013). The PCR conditions were adapted to the qPCR mix (3 mM MgCl_2_, Solis BioDyne, Tartu, Estonia) and a two-step PCR: 10 min at 95°C followed by 45 cycles of 95°C for 15 s and 64°C for 45 s.

The detection of *Fusarium* species was based on the region between primers Fa+7 and Ra+6 of the translation elongation factor gene TEF1 (Karlsson et al., 2016). Probe and primers were selected using the software package DNA star (DNASTAR, Inc., Madison, WI, United States):

S FUS pl probe 5′-CAATAGGAAGCCGC T GAGCTCGGTAAGGGTTC-3,
Fa pl3 forward 5′-TACCCCGCCACTCGAGCG-3′,
Fus pl reverse 5′-TTGAGCTTGTCAAGAACCCAGGCG-3′.

The PCR cycles included 95°C for 10 min followed by 45 cycles of 95°C for 15 s, 65°C for 20 s and 72°C for 30 s.

The quantification of alternaria and fusaria was verified by using DNA standards containing the target region for each primer/probe set prepared from the specific fungal cultures. In the *Alternaria*-specific qPCR, *Fusarium* strains were used as negative control and *vice versa*, *Alternaria* strains were used as negative control for *Fusarium*-specific qPCR. To check the primer specificity, a set of strains was selected from the culture collection of microorganisms of the working group “Fungal Interactions” at the Leibniz Centre for Agricultural Landscape Research Müncheberg comprising *Alternaria* strains of the species-group *A. tenuissima* (GST09t, GH50t, and *At*220) and *A. alternata* (GST37a), which have already been described by Müller et al. (2012) and Kahl et al. (2016) as well as *Fusarium* strains of the species *F. graminearum* (*Fg*23 and *Fg*486) and *F. culmorum* (*Fc*13 and *Fc*493) partly characterized (*Fc*13 and *Fg*23) by Korn et al. (2011). In addition, different strains of plant-associated fungal species were used as negative control: reference strains of *Verticillium* (CBS 130603, CBS 130339, CBS 130340, DSM 12230, and CBS 447.54), of *Gibellulopsis* (CBS 747.83) and of *Trichoderma* spp. (St365), preserved in the culture collection of microorganisms of the working group “Fungal Interactions” at the Leibniz Centre for Agricultural Landscape Research Müncheberg.

#### Analyses of Mycotoxins

Different high performance liquid chromatography (HPLC) methods were applied using a Jasco PU 1580 unit (Jasco Germany GmbH, Pfungstadt, Germany) with degasser, quaternary pump, autosampler and UV/Vis (Jasco UV 2075 Plus) as well as fluorescence detector (Jasco FP 1520) to examine the production of the *Fusarium* mycotoxins deoxynivalenol (DON), nivalenol (NIV), zearalenone (ZON) and alpha- and beta-zearalenol (α- and β-ZOL) as well as the *Alternaria* mycotoxins alternariol (AOH), alternariol monomethyl ether (AME), altenuene (ALT), and tenuazonic acid (TeA). The wheat grains overgrown with bacteria and fungi were ground with a mortar and then extracted to detect the mycotoxins. Detailed extraction and HPLC detection methods for *Fusarium* toxins are described by Müller et al. (2010) and for *Alternaria* toxins by Müller and Korn (2013) and Kahl et al. (2016). Photodiode array detection was performed to control toxin identity. Standard substances of DON, NIV, and ZON were obtained from Sigma-Aldrich Chemie GmbH (Taufkirchen, Germany), AOH, AME, ALT and TeA from Romer Labs Diagnostic GmbH (Tulln, Austria). The toxin detection limits in these grains were 10 ng DON, 20 ng NIV, 1 ng ZON, 2 ng α- and β-ZOL, 50 ng AOH, AME, or ALT, and 100 ng TeA per g of fresh substrate mass. All toxin concentrations were calculated on the dry mass of the substrate (ng g^-1^ DM).

### Statistical Analyses

Microbial data of the density on wheat ears and of the antagonism tests on natural substrate were tested for normal distribution (Kolmogorov–Smirnov test) and homogeneity of variances (Levene test). Differences in the mean values were calculated using the *t*-test for independent variables or the non-parametric Kruskal–Wallis test. Data were reported as significant at *P* < 0.05. All statistical analyses were computed with the SPSS statistical package (v. 22.0; SPSS, IBM, Somers, NY, United States).

## Results

### Cell Density of Fluorescent Pseudomonads on Wheat Ears

At the time of late milk/early dough growth stages, high densities of fluorescent pseudomonads were determined consistently on the wheat ears in all three fields without significant differences (**Supplementary Table S1**). The mean values and standard deviations were as follows: 6.47 ± 0.69 log CFU g^-1^ FM in field 1 with 44 samples, 6.47 ± 0.50 log CFU g^-1^ FM in field 2 (*n* = 67), and 6.56 ± 0.59 log CFU g^-1^ FM in field 3 (*n* = 40).

### Antagonistic Activity of *Pseudomonas* Isolates in Dual Cultures With *F. graminearum* 23, *A. tenuissima* 220, and *Ulocladium* spec. 219

A total of 647 isolates of fluorescent pseudomonads from wheat ears were tested for antagonistic activity against the two phytopathogenic fungi *F. graminearum* 23 (*Fg*23) and *A. tenuissima* 220 (*At*220) as well as the saprophyte *Ulocladium* spec. 219 (*U*219) (**Table 1**). Most isolates showed no antagonistic activity. However, 39% of them inhibited the growth of *U*219, 36% that of *At*220 and 5% that of *Fg*23. Only ten *Pseudomonas* isolates (1.5%) were able to suppress the growth of all three fungal strains (**Supplementary Table S2**). The antagonistic potential in the *Pseudomonas* populations of the three fields was unequally expressed. The proportion of antagonists in the bacterial isolates was highest in field 3 and lowest in field 1 as indicated in the inhibitions of fungi *At*220 and *U*219. Regardless of the field, *Fg*23 was the most robust fungal indicator. Its growth was inhibited by only 5% of all *Pseudomonas* isolates tested. The antagonistic activity of the pseudomonads was partly very different at different sampling points in each of the three fields (**Supplementary Table S3**). There were hot spots of antagonistic bacteria, e.g., at sampling point R50 in field 1 and gaps without them, e.g., at the neighboring point R51.

**Table 1:** Antagonistic activity of fluorescent pseudomonads isolated in three fields in dual culture tests against three fungal indicators.

Field/year	Bacterial Isolates (n)	Antagonists among all isolates from the field (in %)		
		Against *Fg*23	Against *At*220	Against *U*219
Field 1/2015	326	5	22	26
Field 2/2016	159	5	46	45
Field 3/2016	162	5	53	59
				
Total	647	5	36	39

*Data in percent of numbers of isolates tested.*

### Antagonistic Activity of Fluorescent Pseudomonads Against the Genera *Fusarium* and *Alternaria* in Dual Culture Tests

Isolates with striking antagonistic activity against the two fungal “standard” indicators *Fg*23 and *At*220 were selected to test their ability to inhibit the growth of a range of *Alternaria* and *Fusarium* species and strains in the dual culture assay. To be able to better deduce strain-, species- or genus-specific reactions of the fungi, their growth inhibitions were recorded semi-quantitatively. This means that no, low, moderate, and strong inhibitions were distinguished. The fusaria responded strain-specific, while the alternaria responded species-specific, if not even genus-specific (**Tables 2**, **3**). In addition, the sensitivity of the alternaria to the bacterial antagonists was much higher than that of the fusaria, which was reflected in more moderate and strong responses.

**Table 2:** Antagonistic activity of fluorescent pseudomonads against fungi of the genus *Fusarium*.

					Indicators				
	
*Pseudomonas* isolates (species group)^∗^	*Fc*13	*Fc*136	*Fc*358	*Fc*359	*Fg*23	*Fg*355	*Fg*374	*Fe*274	*Fo*10A	*Fs*371
*P. protegens* CHA0	+++	-	+++	+++	++	+++	++	++	++	++
*P. libanensis* 9 (Ia)^#^	-	+++	-	-	++	-	++	+++	++	-
*P. antarctica* 98 (I)	-	++	-	-	++	+	+	++	+	++
*P. rhodesiae* 188 (I)	-	+	-	-	++	-	+	+	++	++
*P.* sp. 262 (I)	+	-	+	+	-	+	++	++	++	+
*P.* sp. 279 (I)	+	-	+	+	+	+	+	+	-	-
*P.* sp. 282 (I)	+	-	++	-	-	+	-	++	+	-
*P.* sp. 339 (II)	-	+++	-	-	+	-	++	++	++	-
*P.* sp. 342 (II)	-	-	-	-	-	-	-	+	++	++
*P.* sp. 346 (II)	-	++	-	-	-	-	+	++	++	-
*P.* sp. 409 (I)	+	-	-	-	-	++	++	+	++	-
*P.* sp. 413 (I)	+	-	-	-	-	+	++	+++	+	++
*P* sp. 418 (I)	+	-	-	-	-	+	++	+++	+	++
*P.* sp. 423 (I)	+	-	-	-	+	+	+	++	-	++
*P.* sp. 429 (I)	++	-	-	-	-	-	-	++	-	-
*P.* sp. 491 (I)	++	-	-	+	+	+	+	+	+	++
*P.* sp. 500 (I)	+	-	-	+	+	++	++	+	+	++
*P.* sp. 516 (III)	-	-	-	-	++	+	+	+	+	++
*P.* sp. 523 (III)	-	++	-	-	++	++	-	+	+	++
*P.* sp. 538 (III)	-	-	-	-	+	++	+	+	-	++

*Fc, Fusarium culmorum; Fg, F. graminearum; Fe, F. equiseti; Fo, F. oxysporum; Fs, F. sporotrichioides. - none, + low, ++ moderate, +++ strong growth inhibition. ^∗^Determined by MALDI-TOF MS; similarity scores within a strain group are higher than 2.3, which are considered to be highly probable species identification. ^#^Reference strain of former studies displayed a score value with strains of group I in the range between 2.0 and 2.2, which is considered to be probable species identification and therefore, it is defined as a subgroup a of group I.*

**Table 3:** Antagonistic activity of fluorescent pseudomonads against fungi of the genus *Alternaria*.

	Indicators													
*Pseudomonas*	*Aa*			*At*				*Ai*		
Isolates (species group)^∗^	CABI^1^	*Aa*GH32	*Aa*GSt37	CABI^1^	*At*St09	*At*H50	*At*220	CBS^2^	*Ai*St01	*Ai*H53
*P. protegens* CHA0	++	++	+++	++	++	++	++	+++	++	+++
*P. libanensis* 9 (Ia)^#^	+++	+++	+++	++	++	+++	++	++	++	++
*P. antarctica* 98 (I)	+	++	++	+	-	+	-	+	+	+
*P. rhodesiae* 188 (I)	+++	+++	+++	+++	+++	+++	++	+++	+++	+++
*P.* sp. 262 (I)	++	++	++	++	++	++	+	++	+++	+++
*P.* sp. 279 (I)	++	++	++	+	++	+	+	++	++	++
*P.* sp. 282 (I)	++	++	++	++	++	++	+	++	++	++
*P.* sp. 339 (II)	+++	+++	+++	++	++	++	++	++	++	+++
*P.* sp. 342 (II)	++	++	++	-	++	-	+++	-	-	++
*P.* sp. 346 (II)	++	+++	+++	++	++	++	++	++	++	++
*P.* sp. 409 (I)	++	++	+++	++	+++	++	++	++	++	+++
*P.* sp. 413 (I)	++	++	++	++	+	+	++	++	++	++
*P.* sp. 418 (I)	++	++	++	++	++	++	++	++	++	++
*P.* sp. 423 (I)	++	++	++	++	++	++	++	++	++	++
*P.* sp. 429 (I)	-	-	++	+	++	+	-	+	+	-
*P*. sp. 491 (I)	++	++	++	++	++	++	++	+++	++	+++
*P.* sp. 500 (I)	++	++	++	+	+++	++	++	+++	+++	++
*P.* sp. 516 (III)	++	++	++	-	++	+	+	++	+	++
*P.* sp. 523 (III)	++	++	++	+	++	+	+	+	+	++
*P.* sp. 538 (III)	+	+	+++	-	++	+	++	+	-	++

*Aa, Alternaria alternata; At, A. tenuissima; Ai, A. infectoria. - none, + low, ++ moderate, +++ strong growth inhibition. ^∗^Determined by MALDI-TOF MS; similarity scores within a strain group are higher than 2.3, which are considered to be highly probable species identification. ^#^Reference strain of former studies displayed a score value with strains of group I in the range between 2.0 and 2.2, which is considered to be probable species identification and therefore, it is defined as a subgroup a of group I. ^*1*^Reference strain from the Centre for Agriculture and Biosciences International in Wallingford, United Kingdom (strain no. CABI 353822 for A. alternata and CABI 352931 for A. tenuissima). ^*2*^Reference strain from the CBS-KNAW collection of the Westerdijk Fungal Biodiversity Institute in Utrecht, Netherlands (strain no. CBS 120149 for A. infectoria).*

### Taxonomic Diversity of Bacterial Isolates

Comparisons of processed spectra of MALDI-TOF MS analyses among each other revealed four groups that showed similarity scores higher than 2.3. These high scores between members of a group indicated a highly probable affiliation at species level. On this basis, four “species groups” were defined, which demonstrated a relatively low diversity of these *Pseudomonas* isolates. Comparison of spectra with the manufacturer’s reference database revealed matches with type strains of different *Pseudomonas* species displaying score values lower than 2.3 but higher than 2.0, which are considered to be probable species identification. Analyses of the ranking table for an isolate, which showed best-matching reference patterns, revealed particularly for isolates of the group I and III that the second match was often a *Pseudomonas* species in the same score range displaying only slightly lower values than for the first match. This result reflects the high phylogenetic relationships between certain species of the genus and therefore, a species affiliation remains in these cases uncertain. In contrast, isolates of the group II and IV revealed a clear difference between match one and two and therefore, a species affiliation is more likely. Thus, strains of group II were identified as *Pseudomonas viridiflava*. Spectra of group IV showed the best match (scores >2.3) with the strain *Pseudomonas koreensis* 2_2 TUB in the manufacturer’s reference database, which differed significantly from the type strain of the species.

However, the proposed species identification enables to affiliate the species groups into phylogenetic groups, which were determined by a multi-locus sequence analysis on the basis of four reference genes (Mulet et al., 2010). The species proposed for group I (*P. fluorescens*, *P. libanensis*, *P. poae*, *P. antarctica*, *P. cedrina*, *P. orientalis*, *P. trivialis*, *P. rhodesiae*, and *P. synxantha*) belong to the *P. fluorescens* subgroup of the genus *Pseudomonas*. The groups II (*P. viridiflava*) and III (*P. congelans*, *P. caricapapayae*, and *P. savastanoi*) belong to the *P. syringae* subgroup. Isolates of group IV can be affiliated to the *P. koreensis* subgroup. This grouping was taken into account in the identification of the isolates (**Tables 2**, **3** and **Supplementary Table S4**).

### Genes Involved in the Biosynthesis of Antibiotics in Relation to the Antagonistic Activity of *Pseudomonas* Isolates

The primers B2BF and BPR4 (Shirzad et al., 2012) amplified a fragment of the *phlD* gene, the key gene in the synthesis of DAPG, from the DNA of the reference strain CHA0. However, such a PCR product was not amplified from the DNA of any of the other reference strains or of the bacterial isolates (**Supplementary Table S4**). On the other hand, at least one of the *phz*, *pltB*, and *prnC* gene fragments, coding for the biosynthesis of PCA, pyoluteorin and pyrrolnitrin was detected in 89 (81%) of 110 isolates tested. These PCR products were unequally distributed among the isolates: The fragment of the *prnC* gene was most often found (in 65% of the isolates analyzed), while a fragment of the *pltB* gene was amplified in 35%, and that of the *phz* gene was amplified in only 11% of the isolates. Fragments of all three, the *phz*, *pltB*, and *prnC* genes, were detected in 7 (6%) of all isolates tested.

A comparison of the antagonistic activity of selected *Pseudomonas* isolates with the detection of genes for the synthesis of antibiotics revealed contradictions: A pronounced inhibition of fungal growth may be associated with the presence of several antibiotic genes, but it does not have to be. The isolates of the *P. fluorescens* subgroup 477, 491, 493, 500, and 508 displaying three of these genes showed moderate or even strong antagonistic activity against at least two of the three test fungi used in this study (**Supplementary Table S4**). Otherwise, no inhibition of fungal growth was detected in the isolates 498 and 600 of the same subgroup, although also carrying the three antibiotic genes. Most of the investigated isolates (77%) belonged to this species group I, which comprised individuals with and without antibiotic encoding genes and whose antagonistic potential ranged from zero to strong. Most of the isolates without antibiotic genes were only lowly or not antagonistic active at all. However, the isolates 409, 418, and 423 of the *P. fluorescens* subgroup as well as *P. viridiflava* 339, 341, 345, 346, and 358 (species group II) were exceptions with higher activity, although not carrying these genes. Species group II showed the highest antagonistic activity at all: 11 of 12 *P. viridiflava* isolates were moderately or even strongly active against at least one of the three fungal indicators, completely independent of key genes presence for antibiotics production.

### Antagonism Test on Natural Substrate

The fungal strains used in this test were selected according to their aggressiveness potential, as reported by Müller et al. (2012). They colonized the wheat grains of the control approaches without bacterial inoculation very densely. This provided the prerequisites for the test of an inhibitory effect by bacterial antagonists.

**FIGURE 1:**
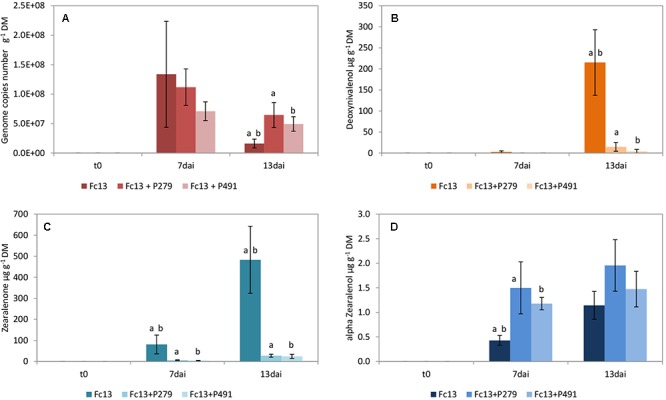
Growth of *Fusarium culmorum* 13 (Fc13) and mycotoxin production on wheat grains. Fungal growth **(A)** as well as the production of deoxynivalenol **(B)**, zearalenone **(C)**, and α-zearalenol **(D)** were influenced by co-inoculation with *Pseudomonas* sp. 279 (P279) and *Pseudomonas* sp. 491 (P491). Samples were taken immediately after inoculating the fungi (t0) and 7 (7dai) as well as 13 (13dai) days after. The values are averages of five replications. Error bars show standard deviation. Values marked with the same letters indicate significant differences at *P* < 0.05.

The growth inhibition of *F. culmorum* 13 on wheat grains by *P.* sp. 279 as well as by *P.* sp. 491 (both of the *P. fluorescens* subgroup) is not statistically verified (**Figure 1A**). The bacterial isolates were differently active in the dual culture assay with low inhibition by *P.* sp. 279 and strong inhibition by *P.* sp. 491 (**Table 2**). Furthermore, the former one contains the gene for pyrrolnitrin production and the latter that for PCA and for pyrrolnitrin (**Supplementary Table S4**). Both bacterial isolates had already reached a high density on the wheat grains 2 days after inoculation, which increased slightly until the seventh day and remained unchanged until the 13th day (**Figure 4A**).

The production of the mycotoxins DON and ZON by *F. culmorum* 13 was strongly reduced without verified differences between the two bacterial inoculants (**Figures 1B,C**). On the other hand, the production of α-ZOL was enhanced under the influence of both bacterial isolates (**Figure 1D**). There was an exception in the case of NIV, which was actually not formed by *F. culmorum* 13 (**Supplementary Table S5**). However, only the inoculation with *P.* sp. 279 enabled the formation of this mycotoxin in low concentrations (4.08 ± 1.29 μg g^-1^ grain dry mass) on day 13.

**FIGURE 2:**
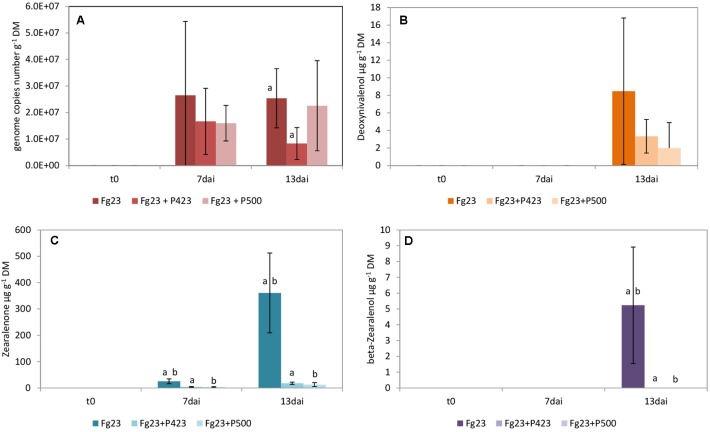
Growth of *Fusarium graminearum* 23 (Fg23) and mycotoxin production on wheat grains. Fungal growth **(A)** as well as the production of deoxynivalenol **(B)**, zearalenone **(C)**, and β-zearalenol **(D)** were influenced by co-inoculation with *Pseudomonas* sp. 423 (P423) and *Pseudomonas* sp. 500 (P500). Samples were taken immediately after inoculating the fungi (t0) and 7 (7dai) as well as 13 (13dai) days after. The values are averages of five replications. Error bars show standard deviation. Values marked with the same letters indicate significant differences at *P* < 0.05.

The growth of *F. graminearum* 23 was significantly inhibited by *P.* sp. 423 on day 13 after inoculation, but not by strain *P.* sp. 500 (**Figure 2A**). The bacterial isolates (both of the *P. fluorescens* subgroup) did not differ in dual culture tests with low antagonistic activity of both (**Table 2**). However, *P.* sp. 500 contains the genes to synthesize the antibiotics PCA, pyoluteorin, and pyrrolnitrin, while P. sp. 423 has none of them (**Supplementary Table S4**). The bacterial strains grew to a maximum cell density in the grains until the second day after inoculation and maintained this density until the 13th day (**Figure 4B**).

The production of mycotoxins by *F. graminearum* 23 and its suppression by the inoculated pseudomonads was distinctly different from that in *F. culmorum* 13. The main mycotoxin produced by *F. graminearum* 23 is ZON (**Figure 2C**). Its high concentration was significantly decreased by inoculation of both bacterial antagonists. DON was detected in low concentrations only on day 13 after inoculation (**Figure 2B**). The suppression of its production by the two *Pseudomonas* isolates could not be statistically ensured. β-ZOL was detected in very low concentrations after 13 days (**Figure 2D**). Its production was almost completely inhibited by both bacterial isolates. The same applied to α-ZOL (**Supplementary Table S6**). Like *F. culmorum* 13, *F. graminearum* 23 did not form NIV, in this case also not in the presence of the bacterial antagonist.

**FIGURE 3:**
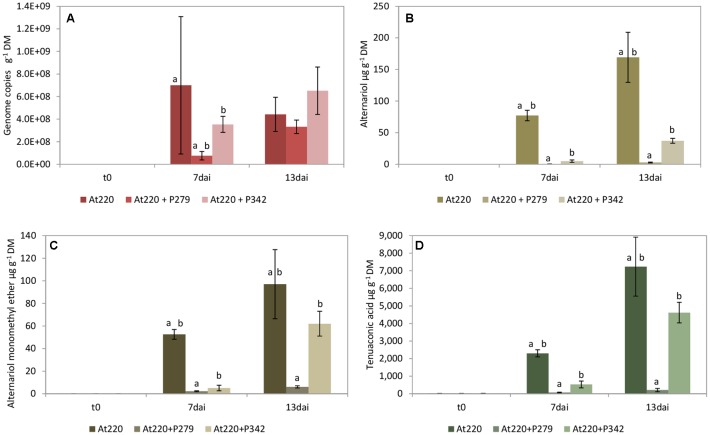
Growth of *Alternaria tenuissima* 220 (At220) and mycotoxin production on wheat grains. Fungal growth **(A)** as well as the production of alternariol **(B)**, alternariol monoethyl ether **(C)**, and tenuazonic acid **(D)** were influenced by co-inoculation with *Pseudomonas* sp. 279 (P279) and *Pseudomonas* sp. 342 (P342). Samples were taken immediately after inoculating the fungi (t0) and 7 (7dai) as well as 13 (13dai) days after. The values are averages of five replications. Error bars show standard deviation. Values marked with the same letters indicate significant differences at *P* < 0.05.

**FIGURE 4:**
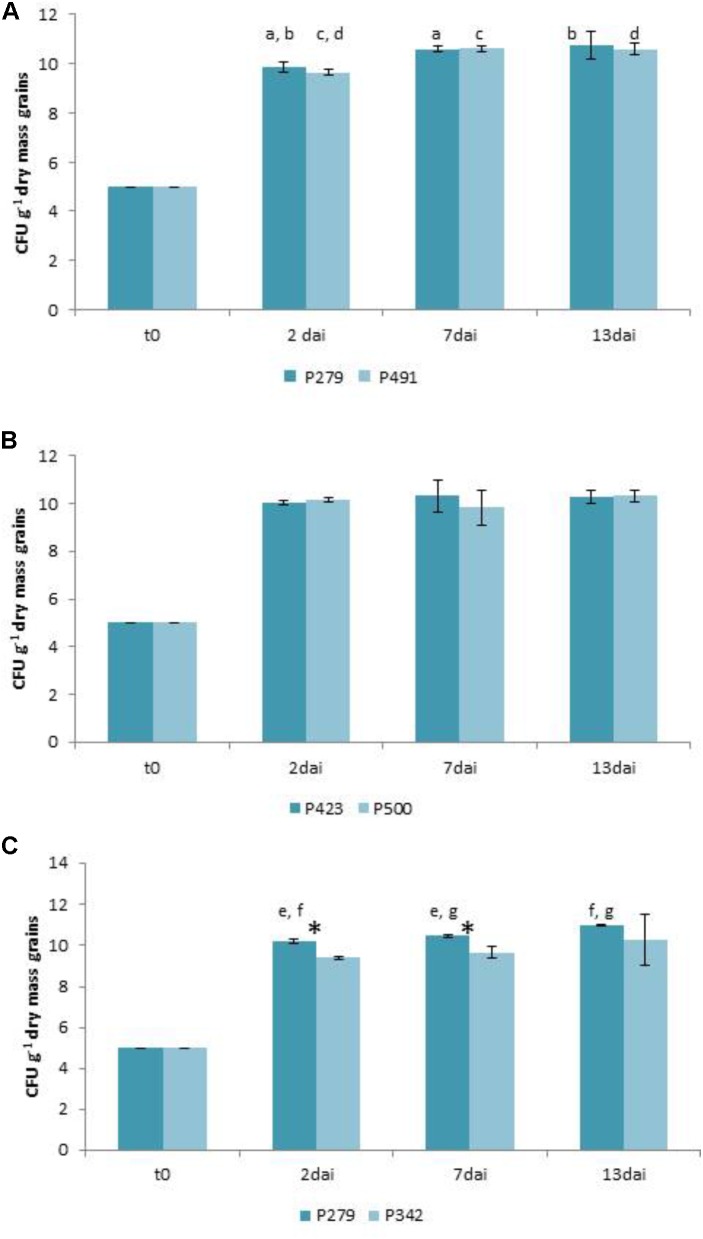
Growth of bacteria in the antagonism tests on natural substrate in colony forming units (CFU) per gram grain (dry mass) on days 2, 7, and 13 after inoculation (dai). Two days after inoculating *Pseudomonas* sp. 279, 342, 423, 491, and 500, respectively, approaches were co-inoculated with *Fusarium culmorum* 13 **(A)**, *F. graminearum* 23 **(B)**, and *Alternaria tenuissima* 220 **(C)**. The values are averages of five replications. Error bars show standard deviations. Values marked with the same letters indicate significant differences in the densities of the same isolate after different incubation intervals at *P* < 0.05. Asterisks indicate different cell densities of the two bacterial isolates at *P* < 0.05.

The growth of *A. tenuissima* 220 was significantly suppressed by *P.* sp. 279 (*P. fluorescens* subgroup) until day 7, but not by *P. viridiflava* 342 and also no longer on day 13 (**Figure 3A**), although the latter was strongly active in the dual culture assay (**Table 3**). Both bacterial isolates harbor the gene coding for pyrrolnitrin (**Supplementary Table S4**). The two isolates grew differently: *P.* sp. 279 developed in these approaches in the same way and at the same level as in combination with *F. culmorum* 13. *P.* sp. 342 already reached its maximum density on the second day, but at a significantly lower level than *P.* sp. 279 (**Figure 4C**).

Among the three fungi used in this test, *A. tenuissima* 220 formed the highest mycotoxin concentrations in the wheat grains with TeA as the predominant compound (**Figure 3D**). The production of the mycotoxins TeA, AOH, and AME was consistently decreased by both antagonists, with *P.* sp. 279 having a stronger inhibitory effect than *P. viridiflava* 342 (**Figures 3B–D**). Also ALT was detected in high concentrations in the approaches with *A. tenuissima* 220: 22.2 ± 1.7 and 173 ± 60.2 μg g^-1^ grain dry mass on days 7 and 13, respectively (**Supplementary Table S7**). Its production was completely inhibited by *P*. sp. 279 and reduced by 92% by *P. viridiflava* 342.

## Discussion

It was our concern to assess the potential antagonistic capabilities of natural *Pseudomonas* populations colonizing wheat heads in first investigations and experiments of an ongoing study. However, it was not our goal to find potential candidates among the bacterial isolates that could be applied as biocontrol agents in the field. The first results of our study indicated that ears of winter wheat at the late milk/early dough stage are colonized with high consistency and at a high density by fluorescent pseudomonads irrespective of the site and the year of investigations. This is particularly a stage of plant development, in which ears are susceptible to infestation by spores of phytopathogenic fungi (Del Ponte et al., 2007).

The assessment of the antagonistic potential of the isolated pseudomonads in the dual culture assay depends not only on their inhibitory effect, but also on the resistance of the fungal indicators used in the test. We based our selection of appropriate indicators on a rating system for fungi, which combines both the length of necrotic lesions on inoculated leaf segments and the *in vitro* mycotoxin production in a cluster analysis (Korn et al., 2011). This analysis assessed the phytopathogenic fungus *F. graminearum* 23 to be highly aggressive. There is also an aggressive potential in *A. tenuissima* 220. However, it is less pronounced than in the *Fusarium* strain (Müller et al., 2012). Fusaria are generally more aggressive than alternaria (Kosiak et al., 2004; Saß et al., 2007). *Ulocladium* is a fungal genus commonly found on herbaceous plants. Species of this genus are similar to those of *Alternaria*, which is also reflected in DNA-based phylogenetic characteristics (Woudenberg et al., 2013, 2015). However, these fungi do not produce mycotoxins. As a saprophyte, the strain *U.* spec. 219 has no aggressive traits and was mostly attacked by fluorescent pseudomonads (**Table 1**). The extent of fungal growth inhibition by antagonistic pseudomonads seems to be dependent on the aggressiveness of these fungi. The results in **Tables 1**–**3** suggest that fluorescent pseudomonads have less potential to affect highly aggressive fungi.

Surprisingly, a fragment of the *phlD* gene responsible for the production of the DAPG, which is considered to be the most promising of the known *Pseudomonas* antibiotics regarding suppression of phytopathogenic fungi (McSpadden Gardener, 2007; Weller et al., 2007; Madloo et al., 2013), was not found in the DNA of any of the pseudomonads isolated from the phyllosphere of wheat in this study. This is in contrast to bacteria of this genus isolated from the rhizosphere of crop plants (Shirzad et al., 2012; Funnell-Harris et al., 2013). But, it is in accordance with findings of Berg et al. (2005). The authors isolated 68 strains of the species *P. putida* with antagonistic activity against *Verticillium dahlia* and/or *Rhizoctonia solani* from potato plants. Most of them (47 strains) originated from the endorhiza, 12 from the rhizosphere, and 9 from the phyllosphere. The *phlD* gene was detected in 44 endorhiza isolates, in 11 of the rhizosphere, but not in any of the phyllosphere isolates. However, Imperiali et al. (2017) most recently investigated the relationships between pathogen resistance in wheat and the abundance as well as expression of the antimicrobial genes for DAPG, phenazines, and pyrrolnitrin on roots in diverse Swiss agricultural soils. Because of complex interactions depending on the host-pathogen system and the soil composition, these relationships could not be generally positively or negatively correlated. The authors concluded that the abundance of pseudomonads producing antimicrobial metabolites may, in fact, be less important for disease suppression than previously hypothesized.

The results of the present study suggest that fluorescent pseudomonads can have an antagonistic effect that is not necessarily associated with the antibiotics that were searched for here. Several reports on plant-associated pseudomonads indicated also a role of siderophores, lytic enzymes, hydrogen cyanide and ammonia as well as organic volatiles in the inhibition of fungal phytopathogens (Alimi et al., 2012; Loper et al., 2012; Müller et al., 2013; Zhang et al., 2014; De Vrieze et al., 2015; Yasmin et al., 2016). Wagner et al. (2018) most recently examined *Pseudomonas* strains to identify the gene clusters that are responsible for antagonism against oomycetes by transposon mutagenesis combined with high throughput sequencing and antagonism tests. As a result of these in-depth investigations, the expression of a specific siderophore was determined. Additionally, diverse antagonistic profiles could be exhibited under certain environmental conditions suggesting that more than one compound was produced that effectively inhibited pathogens. Bailly and Weisskopf (2017) recently published a review on the volatilomes of plant-associated bacteria, underlining the particular importance of volatiles in the antagonism of pseudomonads against *Phytophthora infestans* and other phytopathogens including alternaria and fusaria. The already mentioned inorganic compounds were supplemented by hydrogen sulfide, and among the organic volatiles they indicated 1-undecene and dimethyl disulfide as the most prominent. The authors also addressed the question of what sense volatiles make in the environment, and even more so in the phyllosphere, at the boundary layer between the plant surface and the airspace. When looking at the micro-scale range, they really do make sense. In closed compartments, such as, e.g., the substomatal chamber, which is preferentially colonized by bacteria and favored for the entry of pathogens, volatiles may reach efficient inhibiting concentrations.

An effective biological control of *Alternaria* and *Fusarium* fungi in cereals should ensure both suppressing the spread of the fungus in the field and preventing the formation of mycotoxins. For laboratory investigations, the *Fusarium* strains *F. culmorum* 13 and *F. graminearum* 23 were selected for their aggressiveness and robustness (Korn et al., 2011) to inoculate wheat grains. The strain *A. tenuissima* 220 represents the *Alternaria* species most frequently found on cereal ears in Central Europe (Kahl et al., 2016) and is characterized by its high potential to produce diverse mycotoxins (Müller et al., 2012). The selection of bacterial antagonists was based on semi-quantitative differences in inhibitory capacity in the dual-culture tests and differences in the spectrum of genes for antibiotic synthesis. However, these criteria do not seem to have had any influence on the results of the experimental approaches to investigate these relationships in inoculated wheat grains. The results led to the assumption that under the influence of antagonistic pseudomonads the growth of phytopathogenic fungi is less inhibited than the formation of mycotoxins.

The production of DON by *F. graminearum* in wheat grains has already been shown to be significantly reduced by biocontrol strains of *Bacillus* in laboratory as well as in field experiments (Zhao et al., 2014; Pan et al., 2015). He et al. (2009) reported that this was also possible by inoculating a *Pseudomonas* strain onto wheat heads in a greenhouse. Our laboratory experiments confirmed the inhibition of DON production by pseudomonads. However, the mode of action of the bacterial antagonists in suppressing the syntheses of mycotoxins seems mysterious. Kim et al. (2008) succeeded in inhibiting the biosynthesis of aflatoxin by adding caffeic acid to the nutrient medium at a concentration that did not yet inhibit the growth of *Aspergillus flavus*. Caffeic acid is a natural phenolic acid, which is toxic to many fungi and acts as an antioxidant. Antioxidants and enzymes with antioxidant activity are produced and expressed, respectively, by plants and microorganisms in oxidative stress. They prevent as radical scavengers the oxidation of other substances and quench reactive oxygen species such as superoxide, hydrogen peroxide, and hydroxyl radicals, which are generated, e.g., as a response of the host plant to pathogen infection (Das and Roychoudhury, 2014). On the other hand, reactive oxygen species stimulate the biosynthesis of mycotoxins (Kim et al., 2008; Atanasova-Penichon et al., 2016). Despite very different structures, most mycotoxins, including those of aspergilli, fusaria, and alternaria, are similarly formed from polyketides and thus ultimately from C_2_ and C_3_ units (Rehm, 1973). These intermediates are highly oxygenated by a β-oxidation process involving the acyl-CoA dehydrogenase (Maggio-Hall et al., 2005). This enzyme was downregulated by caffeic acid in the study of Kim et al. (2008). And as a consequence, the mycotoxin synthesis was disturbed. Atanasova-Penichon et al. (2016) recently reviewed the influence of several plant secondary metabolites in cereals on growth and mycotoxin accumulation of fusaria. The authors concluded that oxidative stress also enhanced the synthesis of *Fusarium* toxins, but antioxidant properties of cereal metabolites, mainly terpenoids and phenylpropanoids, could be primary factors preventing these syntheses. The inhibited syntheses of mycotoxins under the influence of pseudomonads in the current study could possibly also be explained by antioxidant activity. In general, all species of the genus *Pseudomonas* are catalase-positive (Palleroni, 2005). Additionally, superoxide dismutases as well as other antioxidant enzymes and mechanisms in pseudomonads were described by Kim and Park (2014). Furthermore, in view of the great diversity of metabolic capabilities in this genus (Palleroni, 2005), other metabolites not yet known to us are conceivable, which may quench reactive oxygen species and thus suppress the synthesis of mycotoxins. Khan et al. (2006) reported a reduced expression of the *Tri5* gene during *F. culmorum* pathogenesis in wheat suggesting an effect of the inoculated biocontrol agent *Pseudomonas* sp. MKB 158. The *F. culmorum Tri5* gene codes for trichodiene synthase, which catalyzes the first unique enzyme in the pathway of trichothecene mycotoxins. The reason for the reduced enzyme expression in the study of Khan et al. (2006) as well as for the inhibited mycotoxin formations in the experiments of the present study may well be the actions of the inoculated pseudomonads against reactive oxygen species.

The mycotoxin analyses in our experimental approaches on wheat grains revealed that α-ZOL was formed by *F. culmorum* 13 in minimal amount. The production of this mycotoxin was further enhanced by inoculation of the bacteria *P.* sp. 279 and *P.* sp. 491 (**Figure 2D**). The reasons for this remain unclear. It is known that α- and β-ZOL are produced as metabolites of ZON in a small extent by *F. culmorum* and *F. graminearum* on artificial media and in naturally contaminated wheat samples (Placinta et al., 1999; Berthiller et al., 2014). However, this could also be caused by a modification of this mycotoxin by the bacteria. Considering that the estrogenic potential of α-ZOL is estimated to be 3 to 4 times higher than that of ZON (Bottalico et al., 1989), this finding should be further investigated.

A considerable inhibition of the growth of *A. tenuissima* 220 by both inoculated bacteria, but especially by *P.* sp. 279, was accompanied by an equally significant reduction in the formation of toxins by this fungus. To the best of our knowledge, apart from a study on the antagonistic activity of the soil bacterium *P. corrugata* against *A. alternata* (Trivedi et al., 2008), nothing comparable has been published so far.

## Conclusion

Under natural field conditions, wheat ears are colonized with high consistency and at a high density by fluorescent pseudomonads at a stage of development, in which they are susceptible to infestation by spores of phytopathogenic fungi. Among these pseudomonads, there may be individual strains with antagonistic potential against alternaria and fusaria. However, it is not possible to deduce from the available results of the intensive *in vitro* investigations presented here, whether this enables the control of growth and distribution of these pathogenic fungi in a field. Our data indicate that the chance of controlling alternaria by naturally occurring antagonistic pseudomonads seems to be better than in fusaria. Otherwise, the bacterial antagonists in the field are obviously not evenly distributed. There are hot spots and gaps. But, even if the proliferation of fungi in the field is unlikely to be inhibited by naturally occurring pseudomonads, it is conceivable that fluorescent pseudomonads could suppress the formation of *Alternaria* and *Fusarium* toxins and thus minimize losses in crop quality. Investigations in the field are required to confirm this assumption. Perhaps, mycotoxin analyses in field samples with high or low densities of antagonists could further clarify the role of fluorescent pseudomonads in this context.

## Author Contributions

TM and MM conceived, designed, and performed the experiments. MM, SR, UB, and PL conducted special analytic work and contributed reagents, materials and analytical equipment. TM, MM, SR, UB, and PL analyzed the data. TM wrote the manuscript with MM and editing assistance from UB, PL, and SR.

## Conflict of Interest Statement

The authors declare that the research was conducted in the absence of any commercial or financial relationships that could be construed as a potential conflict of interest.
