# Supplementary material for: Antagonistic Potential of Fluorescent Pseudomonads Colonizing Wheat Heads Against Mycotoxin Producing Alternaria and Fusaria
Source: Front Microbiol. 2018 Sep 10;9:2124. doi: 10.3389/fmicb.2018.02124 (PMC6139315; doi:10.3389/fmicb.2018.02124)
Supplement: Supplementary file 1 [file Table_1.DOCX]

| **Table S1.** Densities of fluorescent pseudomonads in field samples in log of colony forming | | | | |
| --- | --- | --- | --- | --- |
| units per g wheat heads (fresh matter), n=151 | | |  |  |
|  |  |  |  |  |
| **Sampling** | **Sites** |  | **Sample** |  |
| **date** | **Field/village** | **GPS data** | **no.** | **log CFU/g** |
|  |  |  |  |  |
| June 30, 2015 | Field 1/Raakow | X_Coord 408066,50977 | 2 | 7.37 |
|  |  | Y_Coord 5912493,88704 | 3 | 7.31 |
|  |  |  | 4 | 6.25 |
|  |  |  | 5 | 6.88 |
|  |  |  | 8 | 7.49 |
|  |  |  | 9 | 5.46 |
|  |  |  | 10 | 7.01 |
|  |  |  | 11 | 7.58 |
|  |  |  | 12 | 7.55 |
|  |  |  | 14 | 7.68 |
|  |  |  | 15 | 6.32 |
|  |  |  | 16 | 6.41 |
|  |  |  | 17 | 6.88 |
|  |  |  | 18 | 5.58 |
|  |  |  | 20 | 6.26 |
|  |  |  | 21 | 6.58 |
|  |  |  | 22 | 6.87 |
|  |  |  | 23 | 5.88 |
|  |  |  | 24 | 7.14 |
|  |  |  | 26 | 6.61 |
|  |  |  | 27 | 6.53 |
|  |  |  | 28 | 6.16 |
|  |  |  | 29 | 4.92 |
|  |  |  | 30 | 6.78 |
|  |  |  | 32 | 6.05 |
|  |  |  | 33 | 5.87 |
|  |  |  | 34 | 5.9 |
|  |  |  | 35 | 5.79 |
|  |  |  | 36 | 6.77 |
|  |  |  | 38 | 6.29 |
|  |  |  | 39 | 5.99 |
|  |  |  | 40 | 6.56 |
|  |  |  | 41 | 5.67 |
|  |  |  | 42 | 5.98 |
|  |  |  | 44 | 4.92 |
|  |  |  | 45 | 6.41 |
|  |  |  | 46 | 6.81 |
|  |  |  | 47 | 6.74 |
|  |  |  | 48 | 6.38 |
|  |  |  | 50 | 7.65 |
|  |  |  | 51 | 6.65 |
|  |  |  | 52 | 6.93 |
|  |  |  | 53 | 5.49 |
|  |  |  | 54 | 6.4 |
|  |  |  | n=44 | Mean: 6.47 |
|  |  |  |  | SD: 0.69 |
|  |  |  |  |  |
| June 21, 2016 | Field 2/Bach-Steinfurth | X_Coord 420313,84817 | 1 | 6.33 |
|  |  | Y_Coord 5916253,10291 | 2 | 6.13 |
|  |  |  | 3 | 6.58 |
|  |  |  | 4 | 6.63 |
|  |  |  | 5 | 6.53 |
|  |  |  | 6 | 6.72 |
|  |  |  | 7 | 7.42 |
|  |  |  | 8 | 6.68 |
|  |  |  | 9 | 6.06 |
|  |  |  | 10 | 5.87 |
|  |  |  | 11 | 6.71 |
|  |  |  | 12 | 5.5 |
|  |  |  | 14 | 6.78 |
|  |  |  | 15 | 6.35 |
|  |  |  | 16 | 6.36 |
|  |  |  | 17 | 6.38 |
|  |  |  | 18 | 5.99 |
|  |  |  | 19 | 6.21 |
|  |  |  | 20 | 6.13 |
|  |  |  | 21 | 6.17 |
|  |  |  | 22 | 5.91 |
|  |  |  | 23 | 6.78 |
|  |  |  | 24 | 5.33 |
|  |  |  | 25 | 6.31 |
|  |  |  | 26 | 7.22 |
|  |  |  | 27 | 7.19 |
|  |  |  | 28 | 5.94 |
|  |  |  | 29 | 6.23 |
|  |  |  | 30 | 6.23 |
|  |  |  | 31 | 6.26 |
|  |  |  | 32 | 6,23 |
|  |  |  | 33 | 5.75 |
|  |  |  | 34 | 6.01 |
|  |  |  | 35 | 6.88 |
|  |  |  | 36 | 6.39 |
|  |  |  | 37 | 6.59 |
|  |  |  | 38 | 6.99 |
|  |  |  | 39 | 6.45 |
|  |  |  | 40 | 6.24 |
|  |  |  | 41 | 6.54 |
|  |  |  | 42 | 7.14 |
|  |  |  | 43 | 6.89 |
|  |  |  | 44 | 6.46 |
|  |  |  | 45 | 7.25 |
|  |  |  | 46 | 6.19 |
|  |  |  | 47 | 6.04 |
|  |  |  | 48 | 5.4 |
|  |  |  | 49 | 7.08 |
|  |  |  | 50 | 7.02 |
|  |  |  | 51 | 7.23 |
|  |  |  | 52 | 7.05 |
|  |  |  | 53 | 6.34 |
|  |  |  | 54 | 6.77 |
|  |  |  | 55 | 6.76 |
|  |  |  | 56 | 6.56 |
|  |  |  | 57 | 6.31 |
|  |  |  | 58 | 6.17 |
|  |  |  | 59 | 7.33 |
|  |  |  | 60 | 7.3 |
|  |  |  | 61 | 7.06 |
|  |  |  | 62 | 7.43 |
|  |  |  | 63 | 5.65 |
|  |  |  | 64 | 6.08 |
|  |  |  | 65 | 6.58 |
|  |  |  | 66 | 6.29 |
|  |  |  | 67 | 5.97 |
|  |  |  | 68 | 5.99 |
|  |  |  | n=67 | Mean: 6.47 |
|  |  |  |  | SD: 0.50 |
|  |  |  |  |  |
| June 27, 2016 | Field 3/Arendsee | X_Coord 408812,71676 | 1 | 6.65 |
|  |  | Y_Coord 5909328,33314 | 2 | 7.2 |
|  |  |  | 3 | 6.02 |
|  |  |  | 4 | 6.8 |
|  |  |  | 5 | 6.54 |
|  |  |  | 6 | 6.16 |
|  |  |  | 7 | 5.8 |
|  |  |  | 8 | 6.8 |
|  |  |  | 9 | 7.49 |
|  |  |  | 10 | 6.84 |
|  |  |  | 11 | 7.13 |
|  |  |  | 12 | 7.34 |
|  |  |  | 13 | 6.97 |
|  |  |  | 14 | 4.52 |
|  |  |  | 15 | 6.37 |
|  |  |  | 16 | 6.64 |
|  |  |  | 17 | 7.39 |
|  |  |  | 18 | 7.19 |
|  |  |  | 19 | 6.3 |
|  |  |  | 20 | 6.87 |
|  |  |  | 21 | 6.93 |
|  |  |  | 22 | 6.65 |
|  |  |  | 23 | 6.28 |
|  |  |  | 24 | 7.22 |
|  |  |  | 25 | 6.26 |
|  |  |  | 26 | 6.61 |
|  |  |  | 27 | 6.89 |
|  |  |  | 28 | 6.81 |
|  |  |  | 30 | 5.97 |
|  |  |  | 31 | 5.86 |
|  |  |  | 32 | 6.8 |
|  |  |  | 33 | 6.52 |
|  |  |  | 34 | 6.89 |
|  |  |  | 35 | 5.83 |
|  |  |  | 36 | 6.11 |
|  |  |  | 37 | 5.33 |
|  |  |  | 38 | 6.67 |
|  |  |  | 39 | 6.57 |
|  |  |  | 40 | 6.3 |
|  |  |  | 41 | 6.8 |
|  |  |  | n=40 | Mean: 6.56 |
|  |  |  |  | SD: 0.59 |
|  |  |  |  |  |
