# Supplementary material for: Antagonistic Potential of Fluorescent Pseudomonads Colonizing Wheat Heads Against Mycotoxin Producing Alternaria and Fusaria
Source: Front Microbiol. 2018 Sep 10;9:2124. doi: 10.3389/fmicb.2018.02124 (PMC6139315; doi:10.3389/fmicb.2018.02124)
Supplement: Supplementary file 2 [file Table_2.DOCX]

| **Table S2.** The pseudomonads were isolated in 2015 from 12 samples from field 1 and in 2016 from 5 samples each from fields 2 and 3. | | | | | | | | | | |
| --- | --- | --- | --- | --- | --- | --- | --- | --- | --- | --- |
| The antagonistic activity against fungi was tested in dual culture test with the fungal indicators *Fusarium graminearum* 23 (*Fg*23), | | | | | | | | | | |
| *Alternaria alternata* 220 (*At*220) and *Ulocladium* spec. 219 (*U*219). | | | |  |  |  |  |  |  |  |
| Antagonistic activity was quantified as none (0-3 mm inhibition zone), low (3-5 mm), moderate (5-10 mm) and strong (>10 mm). | | | | | | | | | | |
| The tests were repeated twice. | |  |  |  |  |  |  |  |  |  |
|  |  |  |  |  |  |  |  |  |  |  |
|  |  |  |  |  |  |  |  |  |  |  |
| **Sampling** | **Sites** |  | **Isolate** | **Sample** |  | **Antagonismus** |  | **Antagonismus** |  | **Antagonismus** |
| **dates** | **Field/Village** | **GPS data** | **No.** | **No.** |  | **against *Fg*23** |  | **against *At*220** |  | **against *U*219** |
|  |  |  |  |  |  |  |  |  |  |  |
|  |  |  | CHA0 | Reference |  | ++ |  | ++ |  | +++ |
| June 30, 2015 | Field 1/Raakow | X_Coord 408066,50977 | 201 | R 39 |  | - |  | - |  | + |
|  |  | Y_Coord 5912493,88704 | 202 | R 39 |  | - |  | - |  | - |
|  |  |  | 203 | R 39 |  | - |  | - |  | - |
|  |  |  | 204 | R 39 |  | - |  | + |  | - |
|  |  |  | 205 | R 39 |  | - |  | - |  | - |
|  |  |  | 206 | R 39 |  | - |  | - |  | + |
|  |  |  | 207 | R 39 |  | - |  | - |  | - |
|  |  |  | 208 | R 39 |  | - |  | - |  | + |
|  |  |  | 209 | R 39 |  | - |  | - |  | + |
|  |  |  | 210 | R 39 |  | - |  | - |  | + |
|  |  |  | 211 | R 39 |  | - |  | + |  | - |
|  |  |  | 212 | R 39 |  | - |  | - |  | + |
|  |  |  | 213 | R 39 |  | - |  | + |  | + |
|  |  |  | 214 | R 39 |  | - |  | + |  | - |
|  |  |  | 215 | R 39 |  | - |  | + |  | - |
|  |  |  | 216 | R 39 |  | - |  | + |  | - |
|  |  |  | 217 | R 39 |  | - |  | + |  | - |
|  |  |  | 218 | R 39 |  | - |  | + |  | - |
|  |  |  | 219 | R 39 |  | + |  | - |  | + |
|  |  |  | 220 | R 39 |  | + |  | - |  | - |
|  |  |  | 221 | R 39 |  | - |  | + |  | - |
|  |  |  | 222 | R 39 |  | - |  | - |  | - |
|  |  |  | 223 | R 39 |  | - |  | + |  | - |
|  |  |  | 224 | R 39 |  | - |  | - |  | + |
|  |  |  | 225 | R 39 |  | - |  | + |  | - |
|  |  |  | 225 | R 39 |  | + |  | - |  | - |
|  |  |  | 225 | R 39 |  | - |  | + |  | - |
|  |  |  | 228 | R 39 |  | - |  | - |  | + |
|  |  |  |  |  |  |  |  |  |  |  |
|  |  |  | 229 | R 40 |  | - |  | - |  | - |
|  |  |  | 231 | R 40 |  | + |  | - |  | - |
|  |  |  | 233 | R 40 |  | - |  | - |  | - |
|  |  |  | 234 | R 40 |  | - |  | - |  | - |
|  |  |  | 235 | R 40 |  | - |  | - |  | - |
|  |  |  | 236 | R 40 |  | - |  | - |  | - |
|  |  |  | 237 | R 40 |  | - |  | - |  | - |
|  |  |  | 238 | R 40 |  | - |  | - |  | - |
|  |  |  | 239 | R 40 |  | - |  | - |  | - |
|  |  |  | 240 | R 40 |  | - |  | - |  | - |
|  |  |  | 241 | R 40 |  | - |  | - |  | - |
|  |  |  | 242 | R 40 |  | - |  | - |  | - |
|  |  |  | 243 | R 40 |  | - |  | - |  | - |
|  |  |  | 244 | R 40 |  | - |  | - |  | - |
|  |  |  | 245 | R 40 |  | - |  | - |  | - |
|  |  |  | 246 | R 40 |  | - |  | - |  | - |
|  |  |  | 247 | R 40 |  | - |  | - |  | - |
|  |  |  | 248 | R 40 |  | - |  | - |  | - |
|  |  |  | 249 | R 40 |  | - |  | - |  | - |
|  |  |  | 250 | R 40 |  | - |  | - |  | - |
|  |  |  | 251 | R 40 |  | - |  | - |  | - |
|  |  |  | 252 | R 40 |  | - |  | - |  | - |
|  |  |  | 253 | R 40 |  | - |  | - |  | - |
|  |  |  | 254 | R 40 |  | - |  | - |  | - |
|  |  |  | 255 | R 40 |  | - |  | - |  | - |
|  |  |  | 256 | R 40 |  | - |  | - |  | - |
|  |  |  | 257 | R 40 |  | - |  | - |  | - |
|  |  |  | 258 | R 40 |  | - |  | - |  | - |
|  |  |  | 259 | R 40 |  | - |  | - |  | - |
|  |  |  | 260 | R 40 |  | - |  | - |  | - |
|  |  |  |  |  |  |  |  |  |  |  |
|  |  |  | 261 | R 18 |  | - |  | + |  | ++ |
|  |  |  | 262 | R 18 |  | - |  | + |  | + |
|  |  |  | 263 | R 18 |  | - |  | + |  | + |
|  |  |  | 265 | R 18 |  | - |  | + |  | + |
|  |  |  | 266 | R 18 |  | - |  | + |  | - |
|  |  |  | 267 | R 18 |  | - |  | + |  | + |
|  |  |  | 268 | R 18 |  | - |  | + |  | ++ |
|  |  |  | 269 | R 18 |  | - |  | - |  | ++ |
|  |  |  | 270 | R 18 |  | - |  | - |  | ++ |
|  |  |  |  |  |  | - |  |  |  |  |
|  |  |  | 271 | R 50 |  | - |  | - |  | - |
|  |  |  | 272 | R 50 |  | - |  | + |  | ++ |
|  |  |  | 273 | R 50 |  | - |  | - |  | - |
|  |  |  | 274 | R 50 |  | - |  | - |  | - |
|  |  |  | 276 | R 50 |  | + |  | + |  | ++ |
|  |  |  | 277 | R 50 |  | - |  | - |  | - |
|  |  |  | 278 | R 50 |  | + |  | + |  | + |
|  |  |  | 279 | R 50 |  | + |  | + |  | ++ |
|  |  |  | 280 | R 50 |  | + |  | - |  | - |
|  |  |  | 281 | R 50 |  | - |  | - |  | ++ |
|  |  |  | 282 | R 50 |  | - |  | + |  | ++ |
|  |  |  | 283 | R 50 |  | - |  | - |  | - |
|  |  |  | 284 | R 50 |  | + |  | + |  | ++ |
|  |  |  | 285 | R 50 |  | + |  | + |  | ++ |
|  |  |  | 286 | R 50 |  | - |  | + |  | ++ |
|  |  |  | 287 | R 50 |  | - |  | - |  | - |
|  |  |  | 288 | R 50 |  | - |  | - |  | - |
|  |  |  | 289 | R 50 |  | - |  | - |  | - |
|  |  |  | 290 | R 50 |  | - |  | - |  | - |
|  |  |  | 291 | R 50 |  | - |  | - |  | + |
|  |  |  | 292 | R 50 |  | - |  | - |  | - |
|  |  |  | 293 | R 50 |  | - |  | - |  | ++ |
|  |  |  | 294 | R 50 |  | + |  | - |  | + |
|  |  |  | 295 | R 50 |  | - |  | - |  | - |
|  |  |  | 296 | R 50 |  | - |  | - |  | - |
|  |  |  | 297 | R 50 |  | - |  | - |  | - |
|  |  |  | 298 | R 50 |  | - |  | - |  | + |
|  |  |  | 299 | R 50 |  | - |  | - |  | - |
|  |  |  | 300 | R 50 |  | - |  | - |  | - |
|  |  |  | 301 | R 50 |  | - |  | - |  | - |
|  |  |  | 302 | R 50 |  | - |  | - |  | - |
|  |  |  | 303 | R 50 |  | - |  | - |  | + |
|  |  |  | 304 | R 50 |  | - |  | - |  | + |
|  |  |  | 305 | R 50 |  | - |  | - |  | ++ |
|  |  |  | 306 | R 50 |  | - |  | - |  | - |
|  |  |  |  |  |  |  |  |  |  |  |
|  |  |  | 307 | R 20 |  | - |  | - |  | - |
|  |  |  | 308 | R 20 |  | - |  | - |  | - |
|  |  |  | 309 | R 20 |  | - |  | - |  | - |
|  |  |  | 310 | R 20 |  | - |  | - |  | - |
|  |  |  | 311 | R 20 |  | - |  | - |  | - |
|  |  |  | 312 | R 20 |  | - |  | - |  | - |
|  |  |  | 313 | R 20 |  | - |  | - |  | - |
|  |  |  | 314 | R 20 |  | - |  | - |  | - |
|  |  |  | 315 | R 20 |  | - |  | - |  | - |
|  |  |  | 316 | R 20 |  | - |  | - |  | - |
|  |  |  | 317 | R 20 |  | - |  | - |  | - |
|  |  |  | 318 | R 20 |  | - |  | - |  | - |
|  |  |  | 319 | R 20 |  | + |  | + |  | - |
|  |  |  | 320 | R 20 |  | - |  | - |  | - |
|  |  |  | 321 | R 20 |  | - |  | - |  | - |
|  |  |  | 322 | R 20 |  | - |  | - |  | - |
|  |  |  | 323 | R 20 |  | - |  | - |  | - |
|  |  |  | 324 | R 20 |  | - |  | - |  | - |
|  |  |  | 325 | R 20 |  | - |  | - |  | - |
|  |  |  | 326 | R 20 |  | - |  | - |  | - |
|  |  |  | 327 | R 20 |  | - |  | - |  | - |
|  |  |  | 328 | R 20 |  | - |  | - |  | - |
|  |  |  | 329 | R 20 |  | - |  | - |  | - |
|  |  |  | 330 | R 20 |  | - |  | - |  | - |
|  |  |  | 331 | R 20 |  | - |  | - |  | - |
|  |  |  | 332 | R 20 |  | - |  | - |  | - |
|  |  |  | 333 | R 20 |  | - |  | - |  | - |
|  |  |  | 334 | R 20 |  | - |  | - |  | - |
|  |  |  | 335 | R 20 |  | - |  | - |  | - |
|  |  |  | 336 | R 20 |  | - |  | - |  | - |
|  |  |  |  |  |  |  |  |  |  |  |
|  |  |  | 337 | R 21 |  | - |  | - |  | - |
|  |  |  | 338 | R 21 |  | - |  | - |  | - |
|  |  |  | 339 | R 21 |  | + |  | ++ |  | + |
|  |  |  | 340 | R 21 |  | - |  | - |  | - |
|  |  |  | 341 | R 21 |  | - |  | ++ |  | ++ |
|  |  |  | 342 | R 21 |  | - |  | +++ |  | ++ |
|  |  |  | 343 | R 21 |  | - |  | +++ |  | ++ |
|  |  |  | 344 | R 21 |  | - |  | +++ |  | ++ |
|  |  |  | 345 | R 21 |  | - |  | +++ |  | ++ |
|  |  |  | 346 | R 21 |  | - |  | ++ |  | +++ |
|  |  |  | 347 | R 21 |  | - |  | ++ |  | +++ |
|  |  |  | 348 | R 21 |  | - |  | ++ |  | +++ |
|  |  |  | 349 | R 21 |  | - |  | +++ |  | + |
|  |  |  | 350 | R 21 |  | - |  | ++ |  | ++ |
|  |  |  | 351 | R 21 |  | - |  | - |  | - |
|  |  |  | 352 | R 21 |  | - |  | - |  | + |
|  |  |  | 353 | R 21 |  | - |  | - |  | - |
|  |  |  | 354 | R 21 |  | - |  | ++ |  | ++ |
|  |  |  | 355 | R 21 |  | - |  | ++ |  | ++ |
|  |  |  | 357 | R 21 |  | - |  | - |  | - |
|  |  |  | 358 | R 21 |  | - |  | ++ |  | ++ |
|  |  |  | 359 | R 21 |  | - |  | ++ |  | ++ |
|  |  |  | 360 | R 21 |  | - |  | - |  | - |
|  |  |  | 361 | R 21 |  | - |  | - |  | - |
|  |  |  | 362 | R 21 |  | - |  | - |  | - |
|  |  |  | 363 | R 21 |  | - |  | ++ |  | + |
|  |  |  | 364 | R 21 |  | - |  | ++ |  | ++ |
|  |  |  | 365 | R 21 |  | - |  | + |  | - |
|  |  |  | 366 | R 21 |  | - |  | - |  | - |
|  |  |  | 367 | R 21 |  | - |  | + |  | - |
|  |  |  | 368 | R 21 |  | - |  | - |  | - |
|  |  |  |  |  |  |  |  |  |  |  |
|  |  |  | 401 | R 2 |  | - |  | - |  | - |
|  |  |  | 402 | R 2 |  | - |  | - |  | - |
|  |  |  | 403 | R 2 |  | - |  | - |  | - |
|  |  |  | 404 | R 2 |  | - |  | - |  | - |
|  |  |  | 405 | R 2 |  | - |  | - |  | - |
|  |  |  | 406 | R 2 |  | - |  | - |  | - |
|  |  |  | 407 | R 2 |  | - |  | ++ |  | + |
|  |  |  | 408 | R 2 |  | - |  | ++ |  | ++ |
|  |  |  | 409 | R 2 |  | - |  | ++ |  | ++ |
|  |  |  | 410 | R 2 |  | - |  | - |  | + |
|  |  |  | 411 | R 2 |  | - |  | - |  | - |
|  |  |  | 412 | R 2 |  | - |  | - |  | - |
|  |  |  | 413 | R 2 |  | - |  | ++ |  | + |
|  |  |  | 414 | R 2 |  | - |  | - |  | - |
|  |  |  | 415 | R 2 |  | - |  | - |  | - |
|  |  |  | 416 | R 2 |  | - |  | - |  | - |
|  |  |  | 417 | R 2 |  | - |  | ++ |  | ++ |
|  |  |  | 418 | R 2 |  | - |  | ++ |  | ++ |
|  |  |  | 419 | R 2 |  | - |  | ++ |  | +++ |
|  |  |  | 420 | R 2 |  | - |  | ++ |  | +++ |
|  |  |  | 421 | R 2 |  | - |  | - |  | - |
|  |  |  | 422 | R 2 |  | - |  | - |  | - |
|  |  |  | 423 | R 2 |  | - |  | ++ |  | +++ |
|  |  |  | 424 | R 2 |  | - |  | - |  | - |
|  |  |  | 425 | R 2 |  | - |  | - |  | - |
|  |  |  | 426 | R 2 |  | - |  | - |  | - |
|  |  |  | 427 | R 2 |  | - |  | - |  | - |
|  |  |  | 428 | R 2 |  | - |  | - |  | - |
|  |  |  | 429 | R 2 |  | - |  | - |  | - |
|  |  |  | 430 | R 2 |  | - |  | - |  | - |
|  |  |  | 431 | R 2 |  | - |  | - |  | - |
|  |  |  | 432 | R 2 |  | + |  | ++ |  | - |
|  |  |  | 433 | R 2 |  | - |  | - |  | - |
|  |  |  |  |  |  |  |  |  |  |  |
|  |  |  | 434 | R 3 |  | - |  | - |  | - |
|  |  |  | 435 | R 3 |  | - |  | - |  | - |
|  |  |  | 436 | R 3 |  | - |  | - |  | - |
|  |  |  | 437 | R 3 |  | - |  | - |  | - |
|  |  |  | 438 | R 3 |  | - |  | - |  | + |
|  |  |  | 439 | R 3 |  | - |  | - |  | - |
|  |  |  | 440 | R 3 |  | - |  | - |  | - |
|  |  |  | 442 | R 3 |  | - |  | - |  | - |
|  |  |  | 443 | R 3 |  | - |  | - |  | - |
|  |  |  | 444 | R 3 |  | - |  | - |  | - |
|  |  |  | 445 | R 3 |  | - |  | - |  | - |
|  |  |  | 446 | R 3 |  | - |  | - |  | - |
|  |  |  | 447 | R 3 |  | - |  | - |  | - |
|  |  |  | 448 | R 3 |  | - |  | - |  | - |
|  |  |  | 449 | R 3 |  | - |  | - |  | - |
|  |  |  | 450 | R 3 |  | - |  | - |  | - |
|  |  |  | 451 | R 3 |  | - |  | - |  | - |
|  |  |  | 452 | R 3 |  | - |  | - |  | - |
|  |  |  | 453 | R 3 |  | - |  | - |  | - |
|  |  |  | 454 | R 3 |  | - |  | - |  | - |
|  |  |  | 455 | R 3 |  | - |  | - |  | - |
|  |  |  | 456 | R 3 |  | - |  | - |  | - |
|  |  |  | 457 | R 3 |  | - |  | - |  | - |
|  |  |  | 458 | R 3 |  | - |  | - |  | - |
|  |  |  | 459 | R 3 |  | - |  | - |  | - |
|  |  |  | 460 | R 3 |  | - |  | - |  | - |
|  |  |  | 461 | R 3 |  | - |  | - |  | - |
|  |  |  | 462 | R 3 |  | - |  | - |  | - |
|  |  |  | 463 | R 3 |  | - |  | - |  | - |
|  |  |  |  |  |  | - |  |  |  |  |
|  |  |  | 464 | R 22 |  | - |  | - |  | + |
|  |  |  | 465 | R 22 |  | - |  | - |  | ++ |
|  |  |  | 467 | R 22 |  | - |  | - |  | ++ |
|  |  |  | 468 | R 22 |  | - |  | - |  | - |
|  |  |  | 469 | R 22 |  | - |  | - |  | - |
|  |  |  | 470 | R 22 |  | - |  | ++ |  | ++ |
|  |  |  | 471 | R 22 |  | - |  | - |  | ++ |
|  |  |  | 472 | R 22 |  | - |  | - |  | ++ |
|  |  |  | 473 | R 22 |  | - |  | ++ |  | ++ |
|  |  |  | 475 | R 22 |  | - |  | ++ |  | ++ |
|  |  |  | 476 | R 22 |  | - |  | - |  | - |
|  |  |  | 477 | R 22 |  | - |  | ++ |  | ++ |
|  |  |  | 478 | R 22 |  | - |  | + |  | ++ |
|  |  |  | 479 | R 22 |  | - |  | - |  | ++ |
|  |  |  | 480 | R 22 |  | - |  | ++ |  | ++ |
|  |  |  |  |  |  |  |  |  |  |  |
|  |  |  | 481 | R 23 |  | - |  | - |  | - |
|  |  |  | 482 | R 23 |  | - |  | - |  | - |
|  |  |  | 483 | R 23 |  | - |  | - |  | - |
|  |  |  | 484 | R 23 |  | - |  | - |  | - |
|  |  |  | 485 | R 23 |  | - |  | - |  | - |
|  |  |  | 486 | R 23 |  | - |  | - |  | - |
|  |  |  | 487 | R 23 |  | - |  | + |  | - |
|  |  |  | 488 | R 23 |  | - |  | - |  | - |
|  |  |  | 489 | R 23 |  | - |  | + |  | - |
|  |  |  | 490 | R 23 |  | - |  | - |  | - |
|  |  |  | 491 | R 23 |  | + |  | ++ |  | +++ |
|  |  |  | 492 | R 23 |  | - |  | - |  | - |
|  |  |  | 493 | R 23 |  | - |  | ++ |  | ++ |
|  |  |  | 494 | R 23 |  | - |  | - |  | - |
|  |  |  | 495 | R 23 |  | - |  | + |  | - |
|  |  |  | 496 | R 23 |  | - |  | - |  | - |
|  |  |  | 497 | R 23 |  | - |  | - |  | - |
|  |  |  | 498 | R 23 |  | - |  | - |  | - |
|  |  |  | 499 | R 23 |  | - |  | ++ |  | +++ |
|  |  |  | 500 | R 23 |  | + |  | ++ |  | +++ |
|  |  |  | 501 | R 23 |  | - |  | - |  | - |
|  |  |  | 502 | R 23 |  | - |  | - |  | - |
|  |  |  | 503 | R 23 |  | - |  | - |  | - |
|  |  |  | 504 | R 23 |  | - |  | + |  | +++ |
|  |  |  | 505 | R 23 |  | - |  | - |  | - |
|  |  |  | 506 | R 23 |  | - |  | - |  | - |
|  |  |  | 507 | R 23 |  | - |  | - |  | - |
|  |  |  | 508 | R 23 |  | - |  | ++ |  | ++ |
|  |  |  | 509 | R 23 |  | - |  | - |  | - |
|  |  |  | 510 | R 23 |  | - |  | - |  | - |
|  |  |  | 511 | R 23 |  | - |  | ++ |  | ++ |
|  |  |  |  |  |  |  |  |  |  |  |
|  |  |  | 546 | R 51 |  | - |  | - |  | - |
|  |  |  | 547 | R 51 |  | - |  | - |  | - |
|  |  |  | 548 | R 51 |  | - |  | - |  | - |
|  |  |  | 549 | R 51 |  | - |  | - |  | - |
|  |  |  | 550 | R 51 |  | - |  | - |  | - |
|  |  |  | 551 | R 51 |  | - |  | - |  | - |
|  |  |  | 552 | R 51 |  | - |  | - |  | - |
|  |  |  | 553 | R 51 |  | - |  | - |  | - |
|  |  |  | 554 | R 51 |  | - |  | - |  | - |
|  |  |  | 555 | R 51 |  | - |  | - |  | - |
|  |  |  | 556 | R 51 |  | - |  | - |  | + |
|  |  |  | 557 | R 51 |  | - |  | - |  | - |
|  |  |  | 558 | R 51 |  | - |  | - |  | - |
|  |  |  | 559 | R 51 |  | - |  | - |  | - |
|  |  |  | 560 | R 51 |  | - |  | - |  | - |
|  |  |  | 561 | R 51 |  | - |  | - |  | - |
|  |  |  | 562 | R 51 |  | - |  | - |  | - |
|  |  |  | 563 | R 51 |  | - |  | - |  | ++ |
|  |  |  | 564 | R 51 |  | - |  | - |  | - |
|  |  |  | 565 | R 51 |  | - |  | - |  | - |
|  |  |  | 566 | R 51 |  | - |  | - |  | - |
|  |  |  | 567 | R 51 |  | - |  | - |  | - |
|  |  |  |  |  |  |  |  |  |  |  |
|  |  |  | 568 | R 52 |  | - |  | - |  | - |
|  |  |  | 569 | R 52 |  | - |  | - |  | - |
|  |  |  | 570 | R 52 |  | - |  | - |  | - |
|  |  |  | 571 | R 52 |  | - |  | - |  | - |
|  |  |  | 572 | R 52 |  | - |  | - |  | + |
|  |  |  | 573 | R 52 |  | - |  | - |  | - |
|  |  |  | 574 | R 52 |  | - |  | - |  | - |
|  |  |  | 575 | R 52 |  | - |  | - |  | - |
|  |  |  | 576 | R 52 |  | - |  | - |  | - |
|  |  |  | 577 | R 52 |  | - |  | - |  | - |
|  |  |  | 578 | R 52 |  | - |  | - |  | - |
|  |  |  | 579 | R 52 |  | - |  | - |  | - |
|  |  |  | 580 | R 52 |  | - |  | - |  | - |
|  |  |  | 581 | R 52 |  | - |  | - |  | - |
|  |  |  | 582 | R 52 |  | - |  | - |  | - |
|  |  |  | 583 | R 52 |  | - |  | - |  | - |
|  |  |  | 584 | R 52 |  | - |  | - |  | - |
|  |  |  | 585 | R 52 |  | - |  | - |  | - |
|  |  |  | 586 | R 52 |  | - |  | - |  | - |
|  |  |  | 587 | R 52 |  | - |  | - |  | - |
|  |  |  | 588 | R 52 |  | - |  | - |  | - |
|  |  |  | 589 | R 52 |  | - |  | - |  | - |
|  |  |  | 590 | R 52 |  | - |  | - |  | - |
|  |  |  | 591 | R 52 |  | - |  | - |  | + |
|  |  |  | 592 | R 52 |  | - |  | - |  | - |
|  |  |  | 593 | R 52 |  | - |  | - |  | + |
|  |  |  | 594 | R 52 |  | - |  | - |  | + |
|  |  |  | 595 | R 52 |  | - |  | - |  | - |
|  |  |  | 596 | R 52 |  | - |  | - |  | - |
|  |  |  | 597 | R 52 |  | - |  | - |  | - |
|  |  |  | 598 | R 52 |  | - |  | - |  | + |
|  |  |  | 599 | R 52 |  | - |  | - |  | - |
|  |  |  | 600 | R 52 |  | - |  | - |  | - |
|  |  |  |  |  |  |  |  |  |  |  |
| June 21, 2016 | Field 2/ | X_Coord 420313,84817 | 601 | BS 2 |  | - |  | +++ |  | +++ |
|  | Bach-Steinfurth | Y_Coord 5916253,10291 | 602 | BS 2 |  | - |  | +++ |  | +++ |
|  |  |  | 603 | BS 2 |  | - |  | +++ |  | ++ |
|  |  |  | 604 | BS 2 |  | - |  | +++ |  | ++ |
|  |  |  | 605 | BS 2 |  | - |  | + |  | ++ |
|  |  |  | 606 | BS 2 |  | - |  | +++ |  | ++ |
|  |  |  | 607 | BS 2 |  | - |  | +++ |  | +++ |
|  |  |  | 608 | BS 2 |  | - |  | +++ |  | +++ |
|  |  |  | 609 | BS 2 |  | - |  | ++ |  | ++ |
|  |  |  | 610 | BS 2 |  | - |  | +++ |  | +++ |
|  |  |  | 611 | BS 2 |  | - |  | ++ |  | ++ |
|  |  |  | 612 | BS 2 |  | - |  | +++ |  | +++ |
|  |  |  | 613 | BS 2 |  | - |  | +++ |  | +++ |
|  |  |  | 614 | BS 2 |  | - |  | +++ |  | ++ |
|  |  |  | 615 | BS 2 |  | - |  | +++ |  | +++ |
|  |  |  | 616 | BS 2 |  | - |  | +++ |  | +++ |
|  |  |  | 617 | BS 2 |  | - |  | +++ |  | +++ |
|  |  |  | 618 | BS 2 |  | - |  | +++ |  | +++ |
|  |  |  | 619 | BS 2 |  | - |  | ++ |  | +++ |
|  |  |  | 620 | BS 2 |  | - |  | +++ |  | +++ |
|  |  |  | 621 | BS 2 |  | - |  | +++ |  | +++ |
|  |  |  | 622 | BS 2 |  | - |  | +++ |  | +++ |
|  |  |  | 623 | BS 2 |  | - |  | +++ |  | +++ |
|  |  |  | 624 | BS 2 |  | - |  | +++ |  | +++ |
|  |  |  | 625 | BS 2 |  | - |  | +++ |  | +++ |
|  |  |  | 626 | BS 2 |  | - |  | +++ |  | +++ |
|  |  |  | 627 | BS 2 |  | - |  | ++ |  | + |
|  |  |  | 628 | BS 2 |  | - |  | ++ |  | +++ |
|  |  |  | 629 | BS 2 |  | - |  | +++ |  | +++ |
|  |  |  | 630 | BS 2 |  | - |  | +++ |  | +++ |
|  |  |  | 631 | BS 2 |  | - |  | ++ |  | + |
|  |  |  |  |  |  |  |  |  |  |  |
|  |  |  | 632 | BS 18 |  | - |  | ++ |  | ++ |
|  |  |  | 633 | BS 18 |  | - |  | - |  | - |
|  |  |  | 634 | BS 18 |  | - |  | ++ |  | +++ |
|  |  |  | 635 | BS 18 |  | - |  | - |  | - |
|  |  |  | 636 | BS 18 |  | - |  | - |  | + |
|  |  |  | 637 | BS 18 |  | - |  | ++ |  | +++ |
|  |  |  | 638 | BS 18 |  | ++ |  | - |  | - |
|  |  |  | 639 | BS 18 |  | - |  | - |  | - |
|  |  |  | 640 | BS 18 |  | - |  | - |  | - |
|  |  |  | 641 | BS 18 |  | - |  | - |  | + |
|  |  |  | 642 | BS 18 |  | - |  | - |  | - |
|  |  |  | 643 | BS 18 |  | - |  | - |  | - |
|  |  |  | 644 | BS 18 |  | - |  | - |  | - |
|  |  |  | 645 | BS 18 |  | - |  | - |  | - |
|  |  |  | 646 | BS 18 |  | - |  | - |  | - |
|  |  |  | 647 | BS 18 |  | - |  | - |  | - |
|  |  |  | 648 | BS 18 |  | - |  | - |  | - |
|  |  |  | 649 | BS 18 |  | - |  | - |  | - |
|  |  |  | 650 | BS 18 |  | - |  | ++ |  | + |
|  |  |  | 651 | BS 18 |  | - |  | - |  | - |
|  |  |  | 652 | BS 18 |  | - |  | ++ |  | +++ |
|  |  |  | 653 | BS 18 |  | - |  | - |  | - |
|  |  |  | 654 | BS 18 |  | - |  | +++ |  | +++ |
|  |  |  | 655 | BS 18 |  | - |  | - |  | - |
|  |  |  | 656 | BS 18 |  | - |  | - |  | - |
|  |  |  | 657 | BS 18 |  | - |  | - |  | - |
|  |  |  | 658 | BS 18 |  | - |  | - |  | - |
|  |  |  | 659 | BS 18 |  | + |  | - |  | - |
|  |  |  | 660 | BS 18 |  | + |  | - |  | ++ |
|  |  |  | 661 | BS 18 |  | + |  | + |  | - |
|  |  |  | 662 | BS 18 |  | ++ |  | - |  | - |
|  |  |  | 663 | BS 18 |  | + |  | +++ |  | +++ |
|  |  |  | 664 | BS 18 |  | - |  | +++ |  | ++ |
|  |  |  | 665 | BS 18 |  | - |  | ++ |  | ++ |
|  |  |  |  |  |  |  |  |  |  |  |
| June 27, 2016 | Field3/Arendsee | X_Coord 408812,71676 | 666 | A 25 |  | - |  | - |  | + |
|  |  | Y_Coord 5909328,33314 | 667 | A 25 |  | - |  | - |  | ++ |
|  |  |  | 668 | A 25 |  | - |  | - |  | - |
|  |  |  | 669 | A 25 |  | + |  | - |  | ++ |
|  |  |  | 670 | A 25 |  | + |  | +++ |  | +++ |
|  |  |  | 671 | A 25 |  | - |  | - |  | ++ |
|  |  |  | 672 | A 25 |  | - |  | - |  | - |
|  |  |  | 673 | A 25 |  | - |  | - |  | - |
|  |  |  | 674 | A 25 |  | - |  | - |  | - |
|  |  |  | 675 | A 25 |  | - |  | - |  | + |
|  |  |  | 676 | A 25 |  | - |  | - |  | - |
|  |  |  | 677 | A 25 |  | - |  | + |  | ++ |
|  |  |  | 678 | A 25 |  | - |  | + |  | - |
|  |  |  | 679 | A 25 |  | - |  | - |  | - |
|  |  |  | 680 | A 25 |  | - |  | - |  | - |
|  |  |  | 681 | A 25 |  | - |  | - |  | - |
|  |  |  | 682 | A 25 |  | - |  | - |  | - |
|  |  |  | 683 | A 25 |  | + |  | - |  | - |
|  |  |  | 684 | A 25 |  | + |  | + |  | - |
|  |  |  | 685 | A 25 |  | + |  | + |  | - |
|  |  |  | 686 | A 25 |  | + |  | - |  | - |
|  |  |  | 687 | A 25 |  | - |  | - |  | + |
|  |  |  | 688 | A 25 |  | - |  | - |  | - |
|  |  |  | 689 | A 25 |  | - |  | + |  | - |
|  |  |  | 690 | A 25 |  | - |  | - |  | - |
|  |  |  | 691 | A 25 |  | - |  | - |  | - |
|  |  |  | 692 | A 25 |  | - |  | - |  | - |
|  |  |  | 693 | A 25 |  | - |  | - |  | - |
|  |  |  | 694 | A 25 |  | - |  | - |  | - |
|  |  |  | 695 | A 25 |  | - |  | - |  | - |
|  |  |  | 696 | A 25 |  | - |  | - |  | - |
|  |  |  | 697 | A 25 |  | - |  | - |  | - |
|  |  |  | 698 | A 25 |  | - |  | - |  | - |
|  |  |  |  |  |  |  |  |  |  |  |
|  |  |  | 699 | A 34 |  | - |  | ++ |  | +++ |
|  |  |  | 700 | A 34 |  | - |  | + |  | ++ |
|  |  |  | 701 | A 34 |  | - |  | ++ |  | ++ |
|  |  |  | 702 | A 34 |  | - |  | ++ |  | ++ |
|  |  |  | 703 | A 34 |  | - |  | ++ |  | ++ |
|  |  |  | 704 | A 34 |  | - |  | ++ |  | ++ |
|  |  |  | 705 | A 34 |  | - |  | ++ |  | ++ |
|  |  |  | 706 | A 34 |  | - |  | ++ |  | ++ |
|  |  |  | 707 | A 34 |  | - |  | + |  | ++ |
|  |  |  | 708 | A 34 |  | - |  | ++ |  | ++ |
|  |  |  | 709 | A 34 |  | - |  | ++ |  | +++ |
|  |  |  | 710 | A 34 |  | - |  | ++ |  | ++ |
|  |  |  | 711 | A 34 |  | - |  | ++ |  | +++ |
|  |  |  | 712 | A 34 |  | - |  | ++ |  | +++ |
|  |  |  | 713 | A 34 |  | - |  | + |  | ++ |
|  |  |  | 714 | A 34 |  | - |  | ++ |  | +++ |
|  |  |  | 715 | A 34 |  | - |  | ++ |  | +++ |
|  |  |  | 716 | A 34 |  | - |  | ++ |  | +++ |
|  |  |  | 717 | A 34 |  | - |  | + |  | ++ |
|  |  |  | 718 | A 34 |  | - |  | + |  | ++ |
|  |  |  | 719 | A 34 |  | - |  | ++ |  | ++ |
|  |  |  | 720 | A 34 |  | - |  | + |  | - |
|  |  |  | 721 | A 34 |  | - |  | - |  | + |
|  |  |  | 722 | A 34 |  | - |  | + |  | + |
|  |  |  | 723 | A 34 |  | - |  | + |  | ++ |
|  |  |  | 724 | A 34 |  | - |  | + |  | ++ |
|  |  |  | 725 | A 34 |  | - |  | ++ |  | +++ |
|  |  |  | 726 | A 34 |  | - |  | + |  | +++ |
|  |  |  | 727 | A 34 |  | - |  | + |  | ++ |
|  |  |  | 728 | A 34 |  | - |  | ++ |  | ++ |
|  |  |  | 729 | A 34 |  | - |  | ++ |  | ++ |
|  |  |  | 730 | A 34 |  | - |  | + |  | ++ |
|  |  |  | 731 | A 34 |  | - |  | ++ |  | ++ |
|  |  |  |  |  |  |  |  |  |  |  |
|  |  |  | 732 | A 41 |  | - |  | + |  | ++ |
|  |  |  | 733 | A 41 |  | - |  | ++ |  | ++ |
|  |  |  | 734 | A 41 |  | - |  | ++ |  | ++ |
|  |  |  | 735 | A 41 |  | - |  | ++ |  | ++ |
|  |  |  | 736 | A 41 |  | - |  | ++ |  | ++ |
|  |  |  | 737 | A 41 |  | - |  | ++ |  | ++ |
|  |  |  | 738 | A 41 |  | - |  | ++ |  | ++ |
|  |  |  | 739 | A 41 |  | - |  | + |  | ++ |
|  |  |  | 740 | A 41 |  | - |  | + |  | + |
|  |  |  | 741 | A 41 |  | - |  | + |  | + |
|  |  |  | 742 | A 41 |  | - |  | - |  | ++ |
|  |  |  | 743 | A 41 |  | - |  | + |  | ++ |
|  |  |  | 744 | A 41 |  | - |  | + |  | ++ |
|  |  |  | 745 | A 41 |  | - |  | - |  | +++ |
|  |  |  | 746 | A 41 |  | - |  | + |  | ++ |
|  |  |  | 747 | A 41 |  | - |  | - |  | - |
|  |  |  | 748 | A 41 |  | - |  | - |  | + |
|  |  |  | 749 | A 41 |  | - |  | + |  | ++ |
|  |  |  | 750 | A 41 |  | - |  | - |  | - |
|  |  |  | 751 | A 41 |  | - |  | - |  | ++ |
|  |  |  | 752 | A 41 |  | - |  | + |  | ++ |
|  |  |  | 753 | A 41 |  | - |  | ++ |  | ++ |
|  |  |  | 754 | A 41 |  | - |  | + |  | ++ |
|  |  |  | 755 | A 41 |  | - |  | - |  | - |
|  |  |  | 756 | A 41 |  | - |  | ++ |  | - |
|  |  |  | 757 | A 41 |  | - |  | ++ |  | ++ |
|  |  |  | 758 | A 41 |  | - |  | ++ |  | +++ |
|  |  |  | 759 | A 41 |  | - |  | ++ |  | +++ |
|  |  |  | 760 | A 41 |  | - |  | + |  | ++ |
|  |  |  | 761 | A 41 |  | - |  | + |  | ++ |
|  |  |  | 762 | A 41 |  | - |  | + |  | + |
|  |  |  | 763 | A 41 |  | - |  | - |  | - |
|  |  |  | 764 | A 41 |  | - |  | - |  | + |
|  |  |  |  |  |  |  |  |  |  |  |
|  |  |  | 765 | A 1 |  | - |  | - |  | ++ |
|  |  |  | 766 | A 1 |  | - |  | - |  | - |
|  |  |  | 767 | A 1 |  | - |  | - |  | - |
|  |  |  | 768 | A 1 |  | - |  | - |  | - |
|  |  |  | 769 | A 1 |  | - |  | - |  | - |
|  |  |  | 770 | A 1 |  | - |  | ++ |  | - |
|  |  |  | 771 | A 1 |  | - |  | - |  | ++ |
|  |  |  | 772 | A 1 |  | - |  | - |  | - |
|  |  |  | 773 | A 1 |  | - |  | - |  | - |
|  |  |  | 774 | A 1 |  | - |  | - |  | - |
|  |  |  | 775 | A 1 |  | - |  | - |  | + |
|  |  |  | 776 | A 1 |  | - |  | - |  | - |
|  |  |  | 777 | A 1 |  | - |  | - |  | - |
|  |  |  | 778 | A 1 |  | - |  | - |  | - |
|  |  |  | 779 | A 1 |  | - |  | - |  | - |
|  |  |  | 780 | A 1 |  | - |  | ++ |  | ++ |
|  |  |  | 781 | A 1 |  | - |  | - |  | - |
|  |  |  | 782 | A 1 |  | - |  | - |  | - |
|  |  |  | 783 | A 1 |  | - |  | - |  | + |
|  |  |  | 784 | A 1 |  | - |  | - |  | + |
|  |  |  | 785 | A 1 |  | - |  | - |  | - |
|  |  |  | 786 | A 1 |  | - |  | - |  | + |
|  |  |  | 787 | A 1 |  | - |  | - |  | - |
|  |  |  | 788 | A 1 |  | - |  | - |  | - |
|  |  |  | 789 | A 1 |  | - |  | + |  | ++ |
|  |  |  | 790 | A 1 |  | - |  | - |  | - |
|  |  |  | 791 | A 1 |  | - |  | - |  | - |
|  |  |  | 792 | A 1 |  | - |  | - |  | - |
|  |  |  | 793 | A 1 |  | - |  | - |  | - |
|  |  |  | 794 | A 1 |  | - |  | + |  | ++ |
|  |  |  |  |  |  |  |  |  |  |  |
|  |  |  | 795 | A 9 |  | - |  | + |  | ++ |
|  |  |  | 796 | A 9 |  | - |  | - |  | - |
|  |  |  | 797 | A 9 |  | - |  | + |  | +++ |
|  |  |  | 798 | A 9 |  | - |  | ++ |  | +++ |
|  |  |  | 799 | A 9 |  | - |  | - |  | +++ |
|  |  |  | 800 | A 9 |  | - |  | + |  | +++ |
|  |  |  | 801 | A 9 |  | - |  | ++ |  | +++ |
|  |  |  | 802 | A 9 |  | - |  | ++ |  | + |
|  |  |  | 803 | A 9 |  | - |  | ++ |  | + |
|  |  |  | 804 | A 9 |  | - |  | ++ |  | ++ |
|  |  |  | 805 | A 9 |  | - |  | - |  | + |
|  |  |  | 806 | A 9 |  | - |  | - |  | - |
|  |  |  | 807 | A 9 |  | - |  | - |  | - |
|  |  |  | 808 | A 9 |  | - |  | + |  | ++ |
|  |  |  | 809 | A 9 |  | + |  | + |  | ++ |
|  |  |  | 810 | A 9 |  | + |  | + |  | ++ |
|  |  |  | 811 | A 9 |  | - |  | - |  | + |
|  |  |  | 812 | A 9 |  | - |  | - |  | + |
|  |  |  | 813 | A 9 |  | - |  | ++ |  | ++ |
|  |  |  | 814 | A 9 |  | - |  | ++ |  | +++ |
|  |  |  | 815 | A 9 |  | - |  | + |  | ++ |
|  |  |  | 816 | A 9 |  | - |  | ++ |  | ++ |
|  |  |  | 817 | A 9 |  | - |  | - |  | + |
|  |  |  | 818 | A 9 |  | - |  | + |  | ++ |
|  |  |  | 819 | A 9 |  | - |  | + |  | +++ |
|  |  |  | 820 | A 9 |  | - |  | + |  | +++ |
|  |  |  | 821 | A 9 |  | - |  | - |  | - |
|  |  |  | 822 | A 9 |  | - |  | - |  | + |
|  |  |  | 823 | A 9 |  | - |  | + |  | ++ |
|  |  |  | 824 | A 9 |  | - |  | - |  | - |
|  |  |  | 825 | A 9 |  | - |  | + |  | ++ |
|  |  |  | 826 | A 9 |  | - |  | - |  | - |
|  |  |  | 827 | A 9 |  | - |  | - |  | + |
|  |  |  |  |  |  |  |  |  |  |  |
| June 21, 2016 | Field 2/ | X_Coord 420313,84817 | 828 | BS 19 |  | - |  | - |  | - |
|  | Bach-Steinfurth | Y_Coord 5916253,10291 | 829 | BS 19 |  | - |  | - |  | - |
|  |  |  | 830 | BS 19 |  | - |  | - |  | - |
|  |  |  | 831 | BS 19 |  | - |  | - |  | - |
|  |  |  | 832 | BS 19 |  | - |  | - |  | - |
|  |  |  | 833 | BS 19 |  | - |  | - |  | - |
|  |  |  | 834 | BS 19 |  | - |  | - |  | - |
|  |  |  | 836 | BS 19 |  | - |  | - |  | - |
|  |  |  | 837 | BS 19 |  | - |  | - |  | - |
|  |  |  | 838 | BS 19 |  | - |  | - |  | - |
|  |  |  | 839 | BS 19 |  | - |  | - |  | - |
|  |  |  | 840 | BS 19 |  | - |  | - |  | - |
|  |  |  | 841 | BS 19 |  | - |  | - |  | - |
|  |  |  | 842 | BS 19 |  | - |  | - |  | - |
|  |  |  | 843 | BS 19 |  | - |  | - |  | - |
|  |  |  | 844 | BS 19 |  | - |  | - |  | - |
|  |  |  | 845 | BS 19 |  | - |  | - |  | - |
|  |  |  | 846 | BS 19 |  | - |  | - |  | - |
|  |  |  | 847 | BS 19 |  | - |  | - |  | - |
|  |  |  | 848 | BS 19 |  | - |  | - |  | - |
|  |  |  | 849 | BS 19 |  | - |  | - |  | - |
|  |  |  | 850 | BS 19 |  | - |  | - |  | - |
|  |  |  | 851 | BS 19 |  | - |  | - |  | - |
|  |  |  | 852 | BS 19 |  | - |  | - |  | - |
|  |  |  | 853 | BS 19 |  | - |  | - |  | + |
|  |  |  | 854 | BS 19 |  | - |  | - |  | - |
|  |  |  | 855 | BS 19 |  | + |  | - |  | ++ |
|  |  |  | 856 | BS 19 |  | + |  | - |  | + |
|  |  |  | 857 | BS 19 |  | - |  | - |  | - |
|  |  |  |  |  |  |  |  |  |  |  |
|  |  |  | 858 | BS 21 |  | - |  | - |  | + |
|  |  |  | 859 | BS 21 |  | - |  | - |  | - |
|  |  |  | 860 | BS 21 |  | - |  | - |  | - |
|  |  |  | 861 | BS 21 |  | - |  | - |  | - |
|  |  |  | 862 | BS 21 |  | - |  | - |  | - |
|  |  |  | 863 | BS 21 |  | - |  | - |  | - |
|  |  |  | 864 | BS 21 |  | - |  | - |  | - |
|  |  |  | 865 | BS 21 |  | - |  | - |  | - |
|  |  |  | 866 | BS 21 |  | - |  | - |  | ++ |
|  |  |  | 867 | BS 21 |  | - |  | - |  | +++ |
|  |  |  | 868 | BS 21 |  | - |  | - |  | + |
|  |  |  | 869 | BS 21 |  | - |  | + |  | - |
|  |  |  | 870 | BS 21 |  | - |  | - |  | ++ |
|  |  |  | 871 | BS 21 |  | - |  | - |  | - |
|  |  |  | 872 | BS 21 |  | - |  | - |  | ++ |
|  |  |  | 873 | BS 21 |  | - |  | - |  | - |
|  |  |  | 874 | BS 21 |  | - |  | - |  | ++ |
|  |  |  | 875 | BS 21 |  | - |  | - |  | + |
|  |  |  | 876 | BS 21 |  | - |  | - |  | + |
|  |  |  | 877 | BS 21 |  | - |  | - |  | ++ |
|  |  |  | 878 | BS 21 |  | - |  | - |  | + |
|  |  |  | 879 | BS 21 |  | - |  | - |  | ++ |
|  |  |  | 880 | BS 21 |  | - |  | + |  | ++ |
|  |  |  | 881 | BS 21 |  | - |  | - |  | - |
|  |  |  | 882 | BS 21 |  | - |  | - |  | ++ |
|  |  |  | 883 | BS 21 |  | - |  | - |  | - |
|  |  |  | 885 | BS 21 |  | - |  | - |  | ++ |
|  |  |  | 886 | BS 21 |  | - |  | + |  | - |
|  |  |  | 887 | BS 21 |  | - |  | - |  | - |
|  |  |  | 888 | BS 21 |  | - |  | - |  | - |
|  |  |  | 889 | BS 21 |  | - |  | - |  | + |
|  |  |  |  |  |  |  |  |  |  |  |
|  |  |  | 890 | BS 45 |  | - |  | - |  | - |
|  |  |  | 892 | BS 45 |  | - |  | - |  | + |
|  |  |  | 893 | BS 45 |  | - |  | - |  | - |
|  |  |  | 894 | BS 45 |  | - |  | - |  | - |
|  |  |  | 895 | BS 45 |  | - |  | - |  | - |
|  |  |  | 896 | BS 45 |  | - |  | - |  | - |
|  |  |  | 897 | BS 45 |  | - |  | - |  | - |
|  |  |  | 898 | BS 45 |  | - |  | - |  | - |
|  |  |  | 899 | BS 45 |  | - |  | - |  | - |
|  |  |  | 900 | BS 45 |  | - |  | - |  | - |
|  |  |  | 901 | BS 45 |  | - |  | - |  | - |
|  |  |  | 902 | BS 45 |  | - |  | - |  | - |
|  |  |  | 903 | BS 45 |  | - |  | - |  | - |
|  |  |  | 904 | BS 45 |  | - |  | - |  | - |
|  |  |  | 905 | BS 45 |  | - |  | - |  | + |
|  |  |  | 906 | BS 45 |  | - |  | - |  | - |
|  |  |  | 907 | BS 45 |  | - |  | - |  | - |
|  |  |  | 908 | BS 45 |  | - |  | - |  | - |
|  |  |  | 909 | BS 45 |  | - |  | - |  | - |
|  |  |  | 910 | BS 45 |  | - |  | - |  | - |
|  |  |  | 911 | BS 45 |  | - |  | - |  | ++ |
|  |  |  | 912 | BS 45 |  | - |  | - |  | ++ |
|  |  |  | 913 | BS 45 |  | - |  | - |  | ++ |
|  |  |  | 914 | BS 45 |  | - |  | - |  | - |
|  |  |  | 915 | BS 45 |  | - |  | - |  | + |
|  |  |  | 916 | BS 45 |  | - |  | - |  | - |
|  |  |  | 917 | BS 45 |  | - |  | - |  | + |
|  |  |  | 918 | BS 45 |  | - |  | - |  | - |
|  |  |  | 919 | BS 45 |  | - |  | - |  | + |
|  |  |  | 920 | BS 45 |  | - |  | - |  | ++ |
|  |  |  | 921 | BS 45 |  | - |  | - |  | - |
|  |  |  | 922 | BS 45 |  | - |  | - |  | - |
|  |  |  | 923 | BS 45 |  | - |  | - |  | - |
|  |  |  | 924 | BS 45 |  | - |  | - |  | - |
