# Supplementary material for: Antagonistic Potential of Fluorescent Pseudomonads Colonizing Wheat Heads Against Mycotoxin Producing Alternaria and Fusaria
Source: Front Microbiol. 2018 Sep 10;9:2124. doi: 10.3389/fmicb.2018.02124 (PMC6139315; doi:10.3389/fmicb.2018.02124)
Supplement: Supplementary file 3 [file Table_3.DOCX]

| **Table S3.** Antagonists among the *Pseudomonas* isolates in the samples in percent, detected in dual culture tests. Inhibition zones > 3 mm | | | | | | |
| --- | --- | --- | --- | --- | --- | --- |
| between bacterial colony and fungal mycel on the agar indicated antagonistic activity. The tests were repeated twice. | | | | | |  |
|  |  |  |  |  |  |  |
| **Sampling date** | **Sites** |  | **Sample** | **Proportion of antagonists in the sample (in %)** | | |
|  | **Field/village** | **GPS data** | **No.** | **against *Fg*23** | **against *At*220** | **against *U*219** |
|  |  |  |  |  |  |  |
| June 30, 2015 | Field 1/Raakow | X_Coord 408066,50977 | R 2 | 3.0 | 30.3 | 30.3 |
|  |  | Y_Coord 5912493,88704 | R 3 | 0.0 | 0.0 | 3.4 |
|  |  |  | R 18 | 0.0 | 77.8 | 88.9 |
|  |  |  | R 20 | 3.3 | 3.3 | 0.0 |
|  |  |  | R 21 | 3.2 | 61.3 | 58.1 |
|  |  |  | R 22 | 0.0 | 40.0 | 80.0 |
|  |  |  | R 23 | 6.5 | 32.3 | 22.6 |
|  |  |  | R 39 | 14.3 | 42.9 | 35.7 |
|  |  |  | R 40 | 3.3 | 0.0 | 0.0 |
|  |  |  | R 50 | 20.0 | 22.9 | 45.7 |
|  |  |  | R 51 | 0.0 | 0.0 | 9.1 |
|  |  |  | R 52 | 0.0 | 0.0 | 15.2 |
|  |  |  |  |  |  |  |
| June 21, 2016 | Field 2/ | X_Coord 420313,84817 | BS 2 | 0.0 | 100 | 100 |
|  | Bach-Steinfurth | Y_Coord 5916253,10291 | BS 18 | 17.6 | 29.4 | 35.3 |
|  |  |  | BS 19 | 6.9 | 100 | 10.3 |
|  |  |  | BS 21 | 0.0 | 9.7 | 51.6 |
|  |  |  | BS 45 | 0.0 | 0.0 | 26.5 |
|  |  |  |  |  |  |  |
| June 27, 2016 | Field 3/Arendsee | X_Coord 408812,71676 | A 1 | 0.0 | 13.3 | 70.0 |
|  |  | Y_Coord 5909328,33314 | A 9 | 6.1 | 60.6 | 22.2 |
|  |  |  | A 25 | 18.1 | 22.2 | 24.2 |
|  |  |  | A 34 | 0.0 | 97.0 | 97.0 |
|  |  |  | A 41 | 0.0 | 72.7 | 84.8 |
