# Supplementary material for: Antagonistic Potential of Fluorescent Pseudomonads Colonizing Wheat Heads Against Mycotoxin Producing Alternaria and Fusaria
Source: Front Microbiol. 2018 Sep 10;9:2124. doi: 10.3389/fmicb.2018.02124 (PMC6139315; doi:10.3389/fmicb.2018.02124)
Supplement: Supplementary file 4 [file Table_4.DOCX]

**Table S4.** Selected isolates of fluorescent pseudomonads taxonomically characterized, tested for antagonism against fungal indicators in dual cultures and screened for genes encoding the biosynthesis of antibiotics.

| Antagonistic activity was quantified as none (0-3 mm inhibition zone), low (3-5 mm),  moderate (5-10 mm) and strong (>10 mm). The tests were repeated twice. | | | | | | | | | | | |
| --- | --- | --- | --- | --- | --- | --- | --- | --- | --- | --- | --- |
|  | | |  |  |  |  |  |  |  |  |  |
|  | |  |  | |  |  |  |  |  |  |  |
| Species/species | |  | Antagonismus against | | | |  | Detection of genes | | |  |
| group* | | Isolates | *Fg*23 | | *At*220 | *U*219 |  | *phlD* | *phz* | *pltB* | *prnC* |
| *P. protogens* | | CHA0^1^ | ++ | | ++ | +++ |  | + | - | - | + |
| *P. libanensis*/Ia^#^ | | 9 | + | | +++ | ++ |  | - | - | - | + |
| *P. rhodesia*/I | | 188 | ++ | | ++ | +++ |  | - | - | - | - |
|  | |  |  | |  |  |  |  |  |  |  |
| I | | 295, 306, 307, 324, | - | | - | - |  | - | - | - | - |
|  | | 325, 330, 337, 338, |  | |  |  |  |  |  |  |  |
|  | | 340, 353, 406, 411, |  | |  |  |  |  |  |  |  |
|  | | 434, 450, 577, 579, |  | |  |  |  |  |  |  |  |
|  | | 590, 599 |  | |  |  |  |  |  |  |  |
|  | | 305 | - | | - | ++ |  | - | - | - | - |
|  | | 216 | - | | + | - |  | - | - | - | - |
|  | | 294 | + | | - | + |  | - | - | - | - |
|  | | 278 | + | | + | + |  | - | - | - | - |
|  | | 409, 418 | - | | ++ | ++ |  | - | - | - | - |
|  | | 423 | - | | ++ | +++ |  | - | - | - | - |
|  | | 222, 258, 287, 329, | - | | - | - |  | - | - | - | + |
|  | | 429, 443, 571, 596, |  | |  |  |  |  |  |  |  |
|  | | 229, 242, 252, 253, | - | | - | - |  | - | - | + | + |
|  | | 357, 366, 592, 595, |  | |  |  |  |  |  |  |  |
|  | | 597 |  | |  |  |  |  |  |  |  |
|  | | 498, 600 | - | | - | - |  | - | + | + | + |
|  | | 231, 280 | + | | - | - |  | - | - | - | + |
|  | | 303, 591 | - | | - | + |  | - | - | - | + |
|  | | 281 | - | | - | ++ |  | - | - | - | + |
|  | | 223 | - | | + | - |  | - | - | - | + |
|  | | 267 | - | | + | + |  | - | - | - | + |
|  | | 413 | - | | ++ | + |  | - | - | - | + |
|  | | 272, 282 | - | | + | ++ |  | - | - | - | + |
|  | | 504 | - | | + | +++ |  | - | - | - | + |
|  | | 276, 279 | + | | + | ++ |  | - | - | - | + |
|  | | 555 | - | | - | - |  | - | - | + | - |
|  | | 556 | - | | - | + |  | - | - | + | - |
|  | | 598 | - | | - | + |  | - | + | - | - |
|  | | 351 | - | | - | - |  | - | - | + | + |
|  | | 226 | + | | - | - |  | - | - | + | + |
|  | | 438, 594 | - | | - | + |  | - | - | + | + |
|  | | 269, 293, 465, 563 | - | | - | ++ |  | - | - | + | + |
|  | | 225 | - | | + | - |  | - | - | + | + |
|  | | 262, 263 | - | | + | + |  | - | - | + | + |
|  | | 261, 268, 286 | - | | + | ++ |  | - | - | + | + |
|  | | 419 | - | | ++ | +++ |  | - | - | + | + |
|  | | 285 | + | | + | ++ |  | - | - | + | + |
|  | | 509 | - | | - | - |  | - | + | - | + |
|  | | 473, 511 | - | | ++ | ++ |  | - | + | - | + |
|  | | 491 | + | | ++ | +++ |  | - | + | - | + |
|  | | 477, 493, 508 | - | | ++ | ++ |  | - | + | + | + |
|  | | 500 | + | | ++ | +++ |  | - | + | + | + |
|  | | 489 | - | | + | - |  | - | + | + | + |
| II | | 339 | + | | ++ | + |  | - | - | - | - |
|  | | 341, 358 | - | | ++ | ++ |  | - | - | - | - |
|  | | 345 | - | | +++ | ++ |  | - | - | - | - |
|  | | 346 | - | | ++ | +++ |  | - | - | - | - |
|  | | 363 | - | | ++ | + |  | - | - | - | + |
|  | | 350, 355, 359, 364 | - | | ++ | ++ |  | - | - | - | + |
|  | | 342 | - | | +++ | ++ |  | - | - | - | + |
|  | | 352 | - | | - | + |  | - | - | + | - |
| III | | 271, 277, 296 | - | | - | - |  | - | - | - | - |
|  | | 304 | - | | - | + |  | - | - | - | - |
|  | | 297, 439, 463 | - | | - | - |  | - | - | - | + |
|  | | 283 | - | | - | - |  | - | - | + | + |
|  | | 298 | - | | - | + |  | - | - | - | + |
|  | | 291 | - | | - | + |  | - | - | + | + |
| IV | | 201 | - | | - | + |  | - | - | - | - |
|  | | 224 | - | | - | + |  | - | - | - | + |
|  | | 219 | + | | - | + |  | - | - | + | + |
| *Fg*23 = *Fusarium graminearum* 23, *At*220 = *Alternaria tenuissima* 220, *U*219 = *Ulo-* | | | | | | | | | | |  |
| *cladium* spec. *219*, ^1^) reference strains CHA0, 9 and 188 (s. Material and methods) | | | | | | | | | | |  |
| Antagonistic activity: - none, + low, ++ moderate, +++ strong growth inhibition  * Determined by MALDI-TOF MS; similarity scores within a strain group are higher than 2.3, which are considered to be highly probable species identification.  ^#^ Reference strain of former studies displayed a score value with strains of group I in the range between 2.0 and 2.2, which is considered to be probable species identification and therefore, it is defined as a subgroup a of group I | | | | | | | | | |  |  |
