# Supplementary material for: Antagonistic Potential of Fluorescent Pseudomonads Colonizing Wheat Heads Against Mycotoxin Producing Alternaria and Fusaria
Source: Front Microbiol. 2018 Sep 10;9:2124. doi: 10.3389/fmicb.2018.02124 (PMC6139315; doi:10.3389/fmicb.2018.02124)
Supplement: Supplementary file 8 [file Data_Sheet_1.DOCX]

Supplementary Material

**Antagonistic potential of fluorescent pseudomonads colonizing wheat heads against mycotoxin producing alternaria and fusaria**

Thomas Müller^*^, Silke Ruppel, Undine Behrendt, Peter Lentzsch, Marina E.H. Müller

*** Correspondence:** Corresponding Author: [tmueller@zalf.de](mailto:tmueller@zalf.de)

# Supplementary Figures and Tables

## Supplementary Figures

**Figure S1.** *Pseudomonas* isolates against *Alternaria tenuissima* 220 in culture dual test.

**1.2 Supplementary Tables**

**Table S1.** Densities of fluorescent pseudomonads in field samples.

**Table S2.** All *Pseudomonas* isolates from field 1 in 2015 and from fields 2 and 3 in 2016: detailed data on their antagonistic activity in the dual culture test against three fungal indicators.

**Table S3.** Proportions of antagonists among the isolates in the samples.

**Table S4.** Selected *Pseudomonas* isolates: taxonomy, antagonism, and genes for biosynthesis of antibiotics.

**Table S5.** *Fusarium culmorum* 13 and *Pseudomonas* spec. 279 or *P*. spec. 491 inoculated on wheat grains: microbiological and mycotoxin data.

**Table S6.** *Fusarium graminearum* 23 and *Pseudomonas* spec. 423 or *P*. spec. 500 inoculated on wheat grains: microbiological and mycotoxin data.

**Table S7.** *Alternaria tenuissima* 220 and *Pseudomonas* spec. 279 or *P*. spec. 342 inoculated on wheat grains: microbiological and mycotoxin data.
