# Supplementary figures and images for: Antagonistic Potential of Fluorescent Pseudomonads Colonizing Wheat Heads Against Mycotoxin Producing Alternaria and Fusaria
Source: Front Microbiol. 2018 Sep 10;9:2124. doi: 10.3389/fmicb.2018.02124 (PMC6139315; doi:10.3389/fmicb.2018.02124)

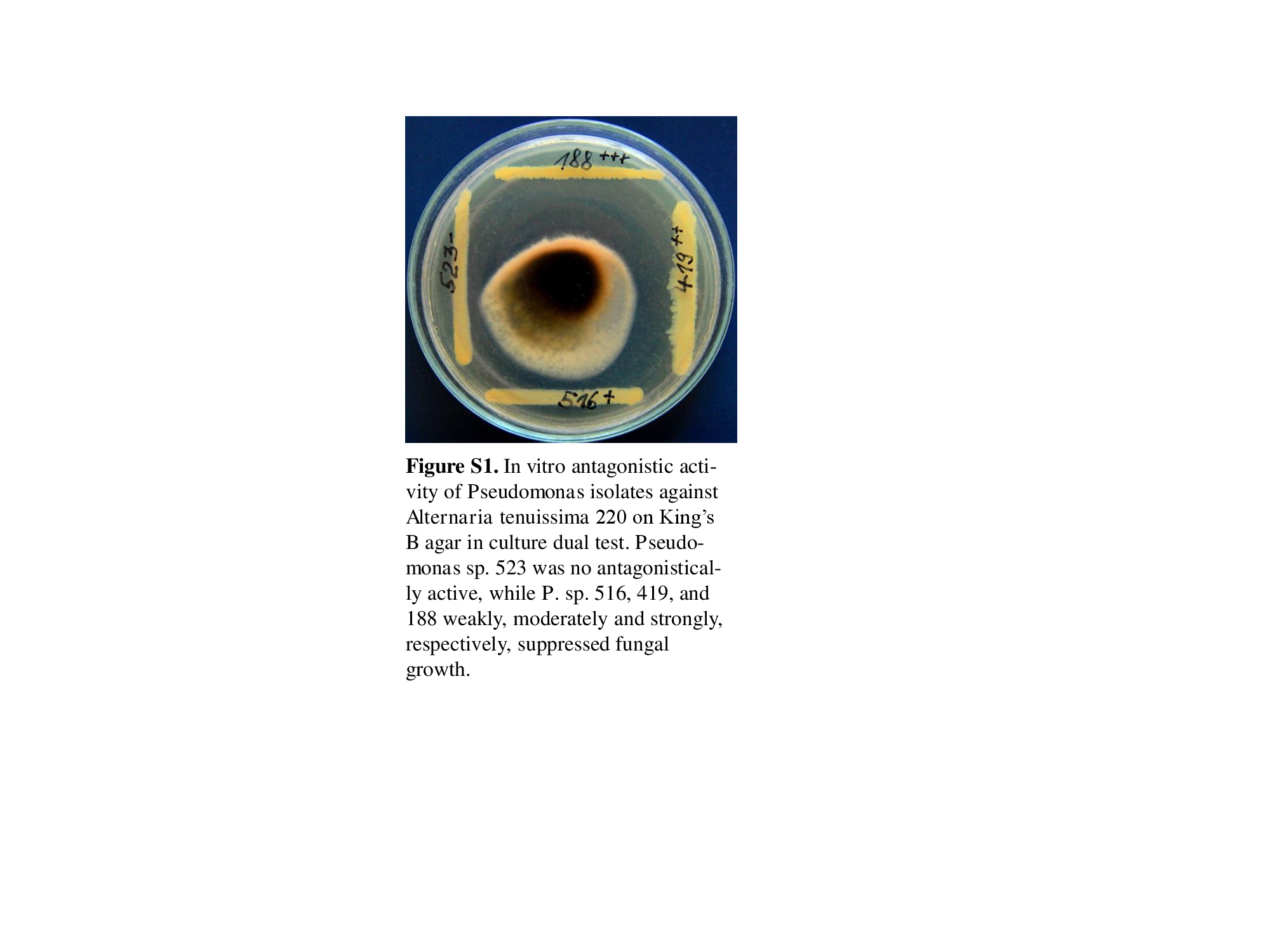

Supplement: Supplementary file 9 [file Image_1.JPEG]
